# Supplementary material for: A single nanophotonic platform for producing circularly polarized white light from non-chiral emitters
Source: Nat Commun. 2024 Nov 30;15:10443. doi: 10.1038/s41467-024-54792-z (PMC11608313; doi:10.1038/s41467-024-54792-z)
Supplement: Supplementary file 1 — Supplementary Information [file 41467_2024_54792_MOESM1_ESM.pdf]

Supplementary Information

A single nanophotonic platform for producing circularly polarized white light from non-chiral emitters

Jose Mendoza-Carreño[1⊥], Simone Bertucci[2,3⊥], Mauro Garbarino[2], Matilde Cirignano[2], Sergio Fiorito[2], Paola Lova[3], Miquel Garriga[1], Maria Isabel Alonso[1] , Francesco Di Stasio[2*] and Agustín Mihi[1]*

1 Institute of Materials Science of Barcelona ICMAB-CSIC, Campus UAB Bellaterra 08193, Spain

2 Photonic Nanomaterials, Istituto Italiano di Tecnologia, 16163, Genova, Italy

3 Dipartimento di Chimica e Chimica Industriale,Università degli Studi di Genova, 16146, Genova, Italy

[⊥]Equal contribution

* E-mail: Francesco.DiStasio@iit.it

* E-mail: amihi@icmab.es

Contents

Supplementary Figures S1-S31

Supplementary note 1: Extra information on the nanophotonic platform

Supplementary note 2: Additional transmission and scanning electron microscopy characterization

Supplementary note 3: Chiral photoluminescence additional information

3.1 Circularly polarized photoluminescence from unpatterned films

3.2 Unpolarized LED excitation photoluminescence

3.3 Time-resolved chiral photoluminescence for all emitters

Supplementary note 1: Extra information on the nanophotonic platform

The experimental ballistic transmittance for both left (red) and right circularly polarized (blue) lights of the chiral nanophotonic platform are shown in **Supplementary Figure 1**. L-(**a**) and R-triskelion (**b**) metasurfaces exhibit opposite behavior for the excitation with circularly polarized light in all the colored shaded areas, thus resulting in an opposite preferential emitted helicity for the probing material disposed atop. Moreover, it is worth noting the difference in chiroptical strength in the green region compared to the other parts of the visible spectrum, elucidating the lower dissymmetry emissions expected for the coupled emitting materials at these wavelengths.

Experimental transmittance of circularly polarized light

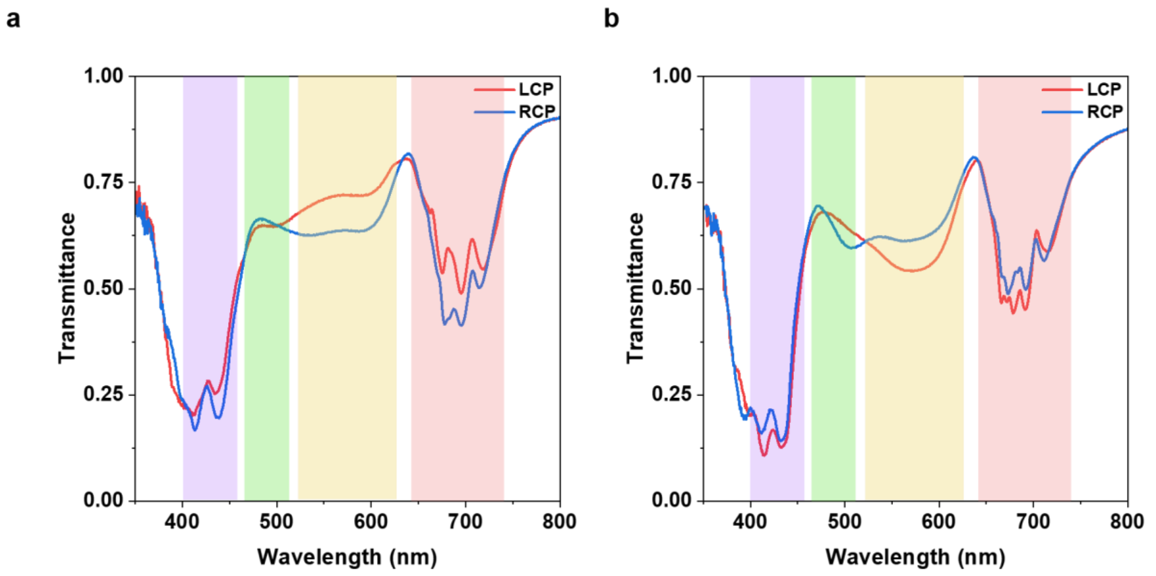

Supplementary Figure 1. Experimental ballistic circularly polarized transmittance for **a**, L- and **b**, R-triskelion metasurfaces. The colored shaded areas indicate the emission band of the probing materials overlapping the chiroptical resonances.

We delve into the effect of rotating the angle between the symmetry axis of the periodic array and the triskelion chiral motif within the unit cell measuring the linear and circular dichroism of the metasurface. As the triskelion motif has C3 symmetry, but the hexagonal array is of higher symmetry (C6), we explore the rotation only within the first 60º. It is worth noting that, for the uncoated triskelion array, only at 45º the motifs overlap, leading to nanofabrication problems. However, for the TiO_2-coated system, large rotation angles within the unit cell lead to a higher volume overlapping, thus resulting in uncertain nanofabrication processes for the coated sample (**Supplementary Figure 2a-e**). We use orthogonal polarizations and calculate the differential transmittance for vertical and horizontal (**Supplementary Figure 2f-j**) and circularly polarized (**Supplementary Figure 2k-o**) light. We observe that the linear dichroism is maximum for the rotation angles of 0 and 45º, and diminishes for 5, 15 and reaching its minimum contribution for 30º. However, the circular

dichroism at this former rotation angle is hindered, as for the case of 15º. Consequently, as a trade-off for minimum linear dichroism contribution and to maximize the circular one, we select the final structure as the one rotated 5º within the unit cell. The further analysis performed from now on corresponds to this rotation angle.

Linear and circular dichroism based on angular displacement

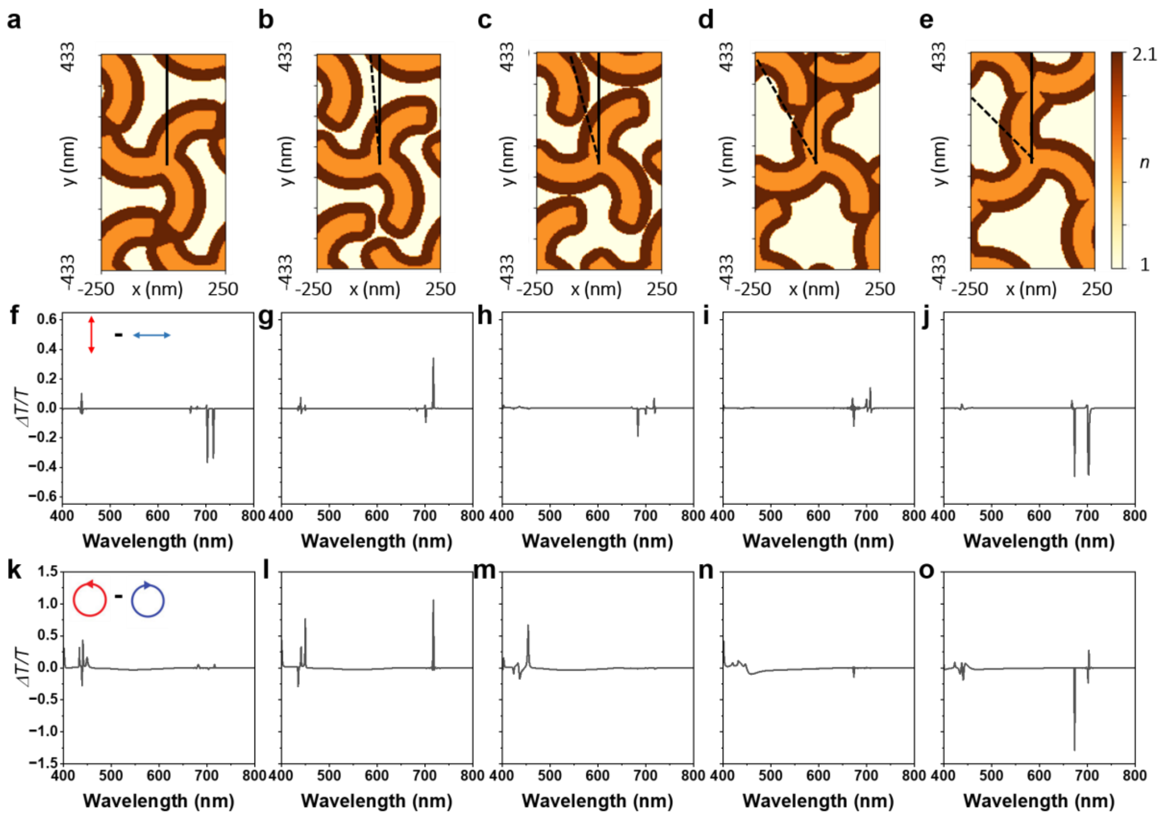

Supplementary Figure 2. (**a-e**) Refractive index of triskelia array unit cell for the rotated angle of 0,5,15,30 and 45º, respectively. (**f-j**) Linear dichroism for the rotated unit cell of triskelion at an angle of 0,5,15,30 and 45º, respectively. (**k-o**) Circular dichroism for the rotated unit cell of triskelion at an angle of 0,5,15,30 and 45º, respectively.

Further insights into the chiral near-field distributions are explored to assess the different spectral resonant origins. To do so, a 500 nm hexagonal lattice triskelion is simulated using commercial FDTD solvers (ANSYS Optics). The structure is composed of a 100 nm thin flat layer and a 160 nm tall triskelion of photoresist. On top of that, 95 nm of high-index TiO_2 coating both as a flat layer and a triskelion-shaped coating is used to enhance light-matter interaction at the nanoscale. A circularly polarized plane-wave-like source is used as excitation by switching the phase delay between $\pm\pi/2$ to excite the near-field chiral modes depending on the incoming polarization, and power monitors measure the fraction of power transmitted through the structure after the interaction. **Supplementary Figure 3** shows the

transmittance difference factor for L- (red) and R-triskelion (blue) where the chiral transitions are in very good agreement with the ones measured experimentally. Sharp and strong chiral transitions are observed both in blue (449 nm) and red (700 nm) wavelengths and a much weaker and broader resonance, centered at 555 nm, is sustained along the green part of the visible spectrum.

Simulated ballistic dissymmetric transmittance factor

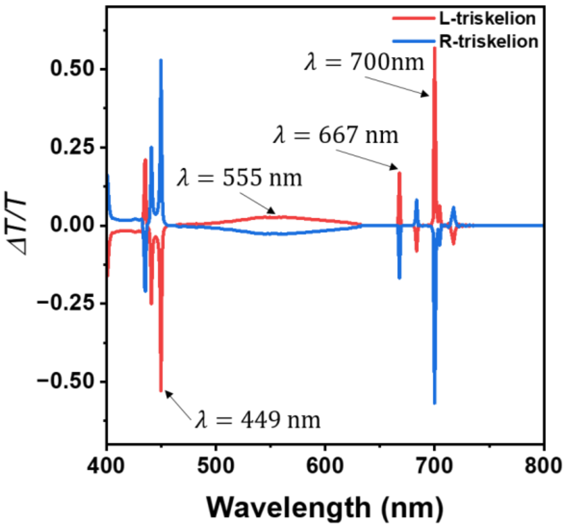

Supplementary Figure 3. Chiral dissymmetry factor $\Delta T/T$ for the simulated structure showing the four different regions of chiral resonances observed experimentally.

We perform FDTD simulations varying the lattice parameter of the triskelia array to identify the collective resonances, which are directly related to the distance between the triskelion motifs (**Supplementary Figure 4**). We observe that the resonances that produce higher fractions of circularly polarized light in the blue and deep red regions are of a diffractive nature, as the dip in transmittance is redshifted for increasing lattice parameters. However, for the broad modes in the central part of the spectrum, no lattice resonances are sustained, thus the origin of these resonances is likely to be local modes, solely related to the triskelion motif. A few guides for the eye lines are drawn to show the increasing number of diffractive resonances when increasing the lattice parameter. For the lowest lattice parameter explored (500 nm), the lattice resonances coincide with the red and blue wavelengths that coincide with the sharp resonances discussed previously.

Simulated ballistic transmittance and dissymmetry as a function of lattice parameter

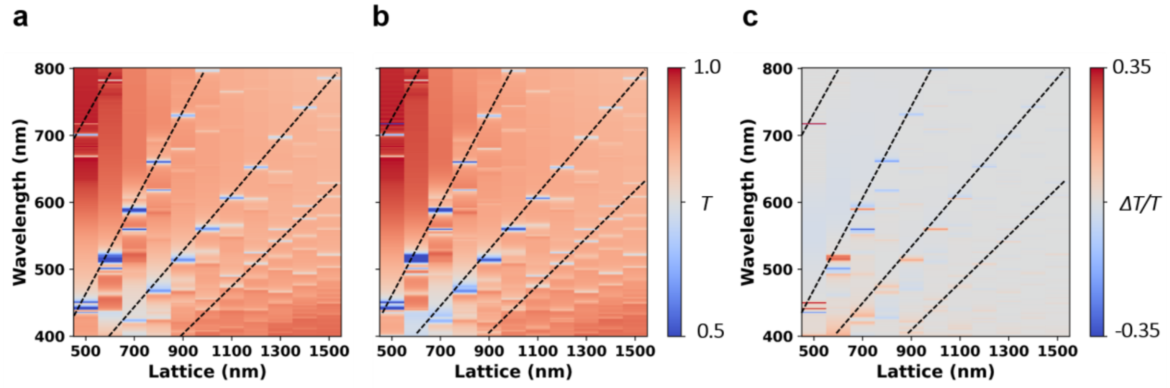

Supplementary Figure 4. Transmittance as a function of lattice parameter for **a**, LCP and **b**, RCP polarizations. **c**, Chiral dissymmetry factor $\Delta T/T$ as a function of the lattice parameter.

Considering the emitters' distribution along the surface observed in the SEM images in the main text, we focus on an intermediate z-plane at 10 nm above the flat layer and before the end of the triskelion structure, where the emitters are located. Spectrally, we observe the electric and magnetic field enhancements of four different emission wavelengths, as specified in **Supplementary Figure 3**. Moreover, we compute the optical chirality factor normalized to that of an RCP plane wave, to account for the helicity of the chiral excited modes depending on the impinging polarization state. The optical chirality is computed as reported elsewhere[1] as:

$$\hat{C} = \frac{C}{C_{\mathrm{RCP}}} = -\frac{c\mu_0}{2}\mathrm{Im}\{\mathbf{E}^* \cdot \mathbf{H}\} \qquad \mathbf{(1)}$$

This normalization carries information on the helicity of the scattered field, thus positive values rotate as RCP waves, whereas negative values show opposite optical rotation and are assigned to LCP waves. Values of optical chirality for propagating fields are comprised between -1 and 1, for perfectly left and right circularly polarized light, respectively. The absence of chirality in the electromagnetic field rotation for linearly polarized light corresponds to optical chirality values of 0. However, non-propagating near-fields can surpass values of 1, hence exceeding the optical rotation of perfect circularly polarized light. When this phenomenon happens, the near fields are referred as *superchiral*[2].

The electric and magnetic field enhancements of an L-triskelion array at $\lambda = 449$ nm are shown in **Supplementary Figure 5**. LCP (first row) and RCP (second row) input polarizations are used as excitation sources. We observe that for RCP impinging light, the electric field enhancement gets up to 15-fold at the interface between air-TiO_2 (**d**), a much stronger interaction compared to an LCP excitation (**a**). Considering now the magnetic field enhancement, a similar phenomenon is observed for RCP (**e**), sustaining magnetic hotspots at the interfaces, hence spatially coinciding both electric and magnetic fields which may

enable higher chiral near-fields compared to LCP (**b**). Finally, we study the normalized optical chirality as specified in equation (**1**) for the scattered field of the resonant mode for both LCP (**c**) and RCP (**f**) impinging excitations. Remarkably, the helicity of the scattered field observed is mainly RCP independently from the incoming polarization state, thus coloring the optical chirality value to positively red located near the arms hotspots for both LCP (**c**) and RCP (**f**). Therefore, emitters placed near the vicinity of these chiral hotspots will undergo an asymmetric emission at this wavelength, as observed in the experimental emission.

Simulated near fields and optical chirality density for λ = 449 nm

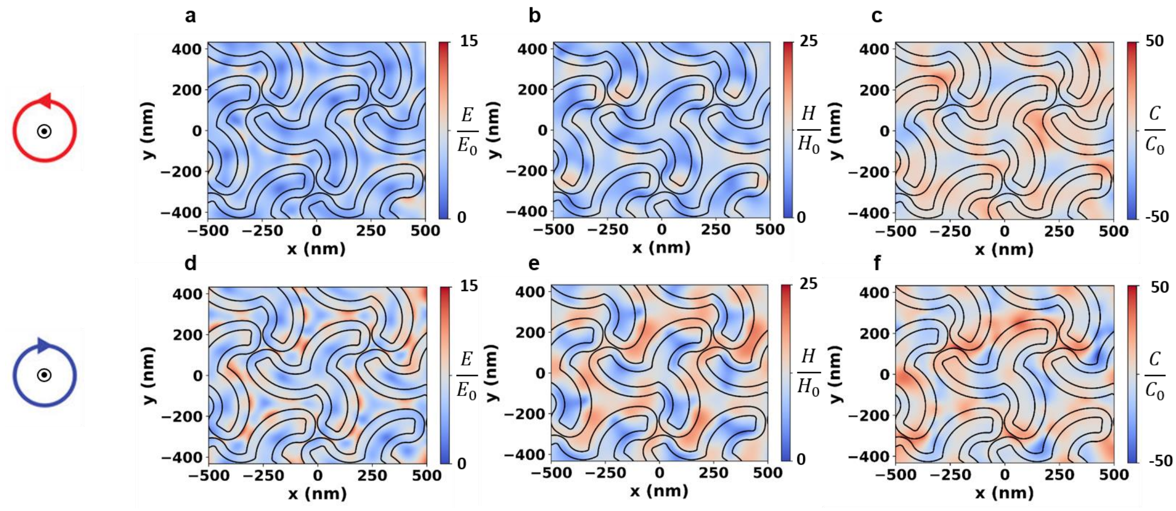

Supplementary Figure 5. Near field enhancements at $\lambda = 449\ nm$ for electric (**a,d**) and magnetic (**b,e**) when Left (first row) and Right circularly polarized (second row) are used as excitation sources. Normalized optical chirality for the resonant near field of LCP (**c**) and RCP (**f**).

Equivalent analysis is performed for the broad weak chiral resonance centered at λ = 555 nm, shown in **Supplementary Figure 6**. The low differences in the transmittance spectra and the absence of peak resonances result in smaller electric (**a,d**) and magnetic field (**b,e**) enhancements and similar behavior of the two enantiomorphic helicities. When observing the optical chirality of both helicities, we observe symmetric distributions of chiral fields with opposite orientations for LCP (**c**) and RCP (**e**), shown as uniform blue and red regions, respectively. Therefore, net optical chirality is weaker, and not distributed along the entire surface, hence resulting in a much less efficient resonance to produce circularly polarized light of emitters at this spectral region.

Simulated near fields and optical chirality density for λ = 555 nm

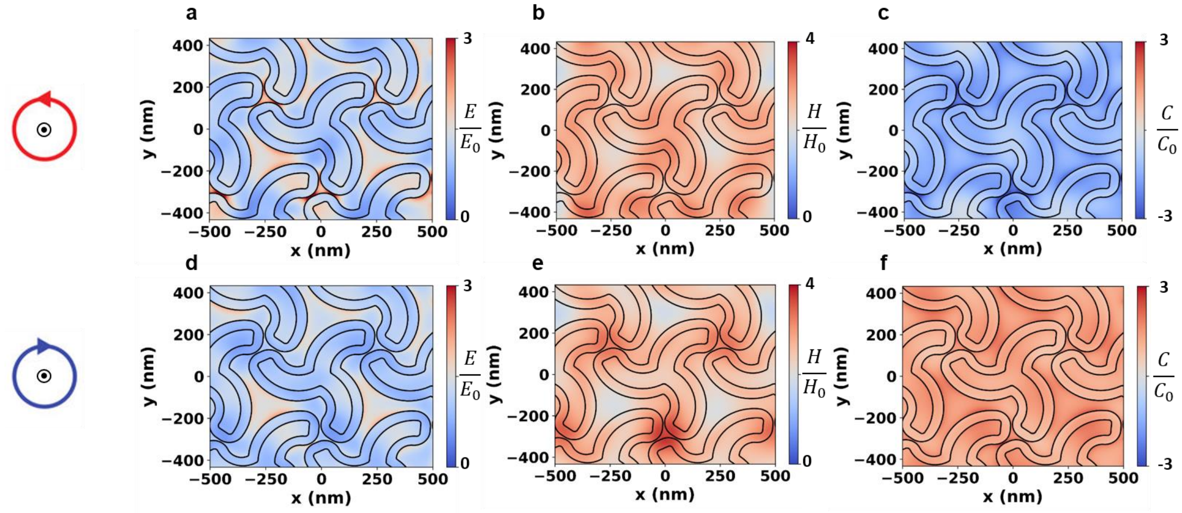

Supplementary Figure 6. Near field enhancements at $\lambda = 555\ nm$ for electric (**a,d**) and magnetic (**b,e**) when Left (first row) and Right circularly polarized (second row) are used as excitation sources. Normalized optical chirality for the resonant near field of LCP (**c**) and RCP (**f**).

The third spectral region studied herein corresponds to a resonant wavelength at λ = 667 nm (**Supplementary Figure 7**). The incoming LCP polarization results in stronger electric and magnetic hotspots (**a,b**). However, the mismatch location between these two fields results in hindered optical chirality density, located mainly within the triskelion motif (**c**). Therefore, the active material cannot access these regions of optically active chirality. On the other hand, even though the incoming RCP polarization results in less intense electric and magnetic hotspots (**d,e**), the spatial matching of these fields exhibits larger values of optical chirality density outside the triskelion motif (**f**). This governs the overall net chirality at this wavelength where the active emitting material will be located.

Simulated near fields and optical chirality density for λ = 667 nm

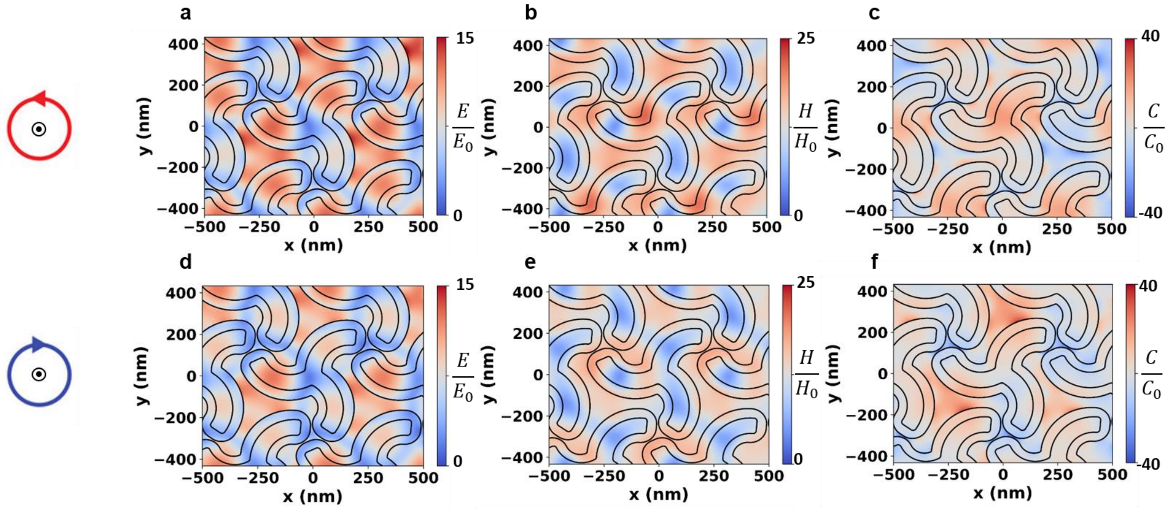

Supplementary Figure 7. Near field enhancements at $\lambda = 667\ nm$ for electric (**a,d**) and magnetic (**b,e**) when Left (first row) and Right circularly polarized (second row) are used as excitation sources. Normalized optical chirality for the resonant near field of LCP (**c**) and RCP (**f**).

Finally, we focus on the last spectral resonance located at λ = 700 nm. At this wavelength, similarly to λ = 449 nm, strong enhancements of both electric and magnetic fields are observed for LCP excitation, shown in **Supplementary Figure 8** panel (**a)** and (**b**), respectively. Besides, both electric and magnetic hotspots are located at the outer area of the triskelion motif and coincide spatially, thus enabling the efficient scattering of the chiral near-field. The normalized values of optical chirality density display enormous values, up to 275-fold compared to circularly polarized plane waves under LCP excitation (**c**). On the other hand, RCP excitation does not sustain great field enhancements compared to its enantiomorphic helicity, hence both electric (**d**) and magnetic (**e**) field enhancements do not provide sufficient opposite optical chirality density (**f**), resulting in large net optical chirality density at this wavelength.

Simulated near fields and optical chirality density for λ = 700 nm

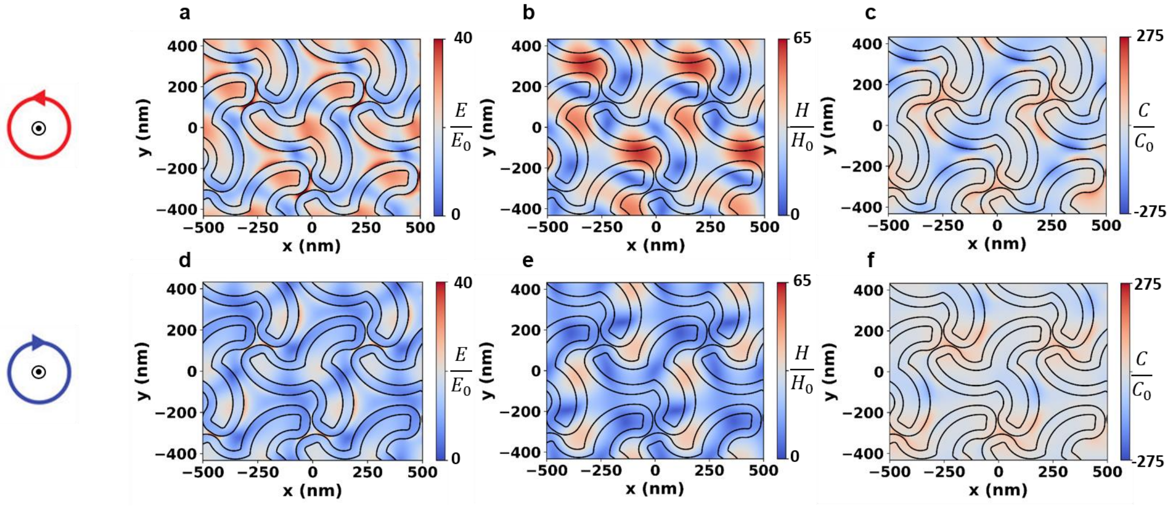

Supplementary Figure 8. Near field enhancements at $\lambda = 700\, nm$ for electric (**a,d**) and magnetic (**b,e**) when Left (first row) and Right circularly polarized (second row) are used as excitation sources. Normalized optical chirality for the resonant near field of LCP (**c**) and RCP (**f**).

An alternative approach to computationally account for the emission dissymmetry for achiral emitters and assuming dipolar transitions as the main contribution is integrating the electric field intensity in the active region[3]. For our structure, the emitters are spin-coated atop of the final structure, therefore the active region is comprised between the top of the TiO_2 flat layer towards the superstrate. However, in the SEM images, we observe that the emitters do not surpass the upper part of the triskelia array, for the concentrations used in this work. Consequently, we integrate the electric field intensity for the injected LCP and RCP polarizations between 200 < z < 280 nm.

$$I_{\mathrm{i}}(x,y) = \frac{\int_l E_{\mathrm{i}}^2(x,y,z)dz}{\iint_l dz} \qquad \textbf{(2)}$$

Supplementary Figure 9 summarizes the calculated integration for the resonant modes studied herein. For the blue-resonant mode at λ = 449 nm, we observe strong differences between the incoming LCP (**a**) and RCP injections (**b**) in the active area, showing a stronger enhancement for the latter case. The broad resonance at λ = 555 nm concentrates the field enhancement at the hotspot where the arms of the triskelia coincide only for one of the polarizations (**c**,**d**). Therefore, this small region accounts for the weak differences observed in the CPE, as only the emitters placed there will contribute to polarized PL. The third resonant wavelength λ = 667 nm shows a similar distribution of the electric field intensity for both polarizations (**e**,**f**). However, a stronger LCP enhancement is observed, resulting in a differential CPE for the emitters at this wavelength. Finally, the resonance at λ = 700 nm shows large differences for the electric field intensity with a much stronger LCP (**g**) than RCP (**h**) incoming excitation. All these results are in agreement with the ones previously discussed for the optical chirality density, where the better in-coupling results in a stronger

enhancement of the electric and magnetic fields and thus using the reciprocity principle one can predict the emitted handedness.

Integrated electric field intensity of simulated near fields at resonant wavelengths

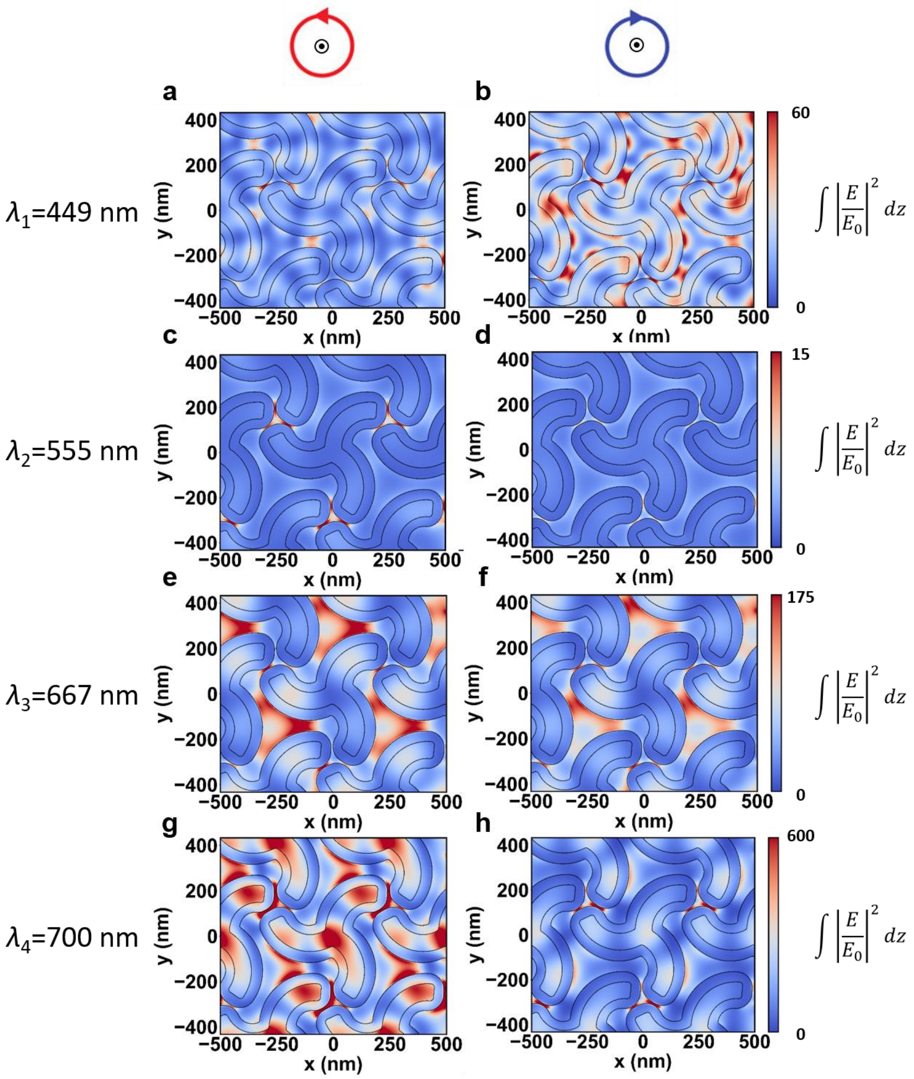

Supplementary Figure 9. Integrated electric field intensity along the propagation z-axis for LCP (left) and RCP (right) waves for the resonant wavelengths **a,b** 449 nm **c,d** 555 nm **e,f** 667 nm and **g,h** 700 nm.

To take into account only the optical chirality density that can be transferred to the active emitting material in the integration process, a mask is applied to the net optical chirality at each spectral region. The net optical chirality is computed as the sum of both optical chirality densities for LCP and RCP injections. This value informs about the spatial regions where the induced optical chirality is not compensated thus resulting in optically active chiral areas that can be transferred to CPE.

Integrating mask used for filtering

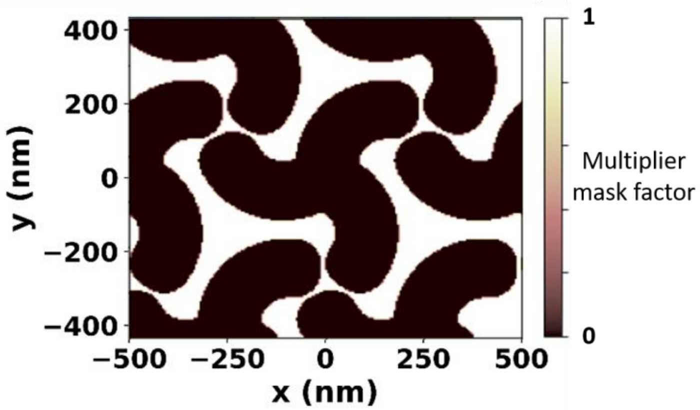

Supplementary Figure 10. Mask applied to the optical chirality density for the active region integration along the propagation axis.

We compute the excess of optical chirality by integrating the values obtained for each z-plane. This informs about the net value of optical chirality as a function of the z-axis distance for the entire unit cell. To do so, we compute the point-to-point multiplication of the net optical chirality density values with the mask previously discussed, resulting in the integration of the regions accessible for the active emitting material placed atop. The integration is computed as:

$$\Delta\hat{C}(z) = \frac{\iint_S \Delta\hat{C}(x,y,z)\cdot F(x,y)dxdy}{\iint_S dxdy} = \frac{\iint_S \Delta\hat{C}(x,y,z)dxdy \cdot F(x,y)}{S} \qquad (3)$$

Where $\Delta\hat{C}(x,y,z)$ indicates the excess of optical chirality as a function of the position (x,y,z), $F(x,y)$ is the mask function and S is the unit cell area for integration.

The active material is deposited just atop the interface between the flat TiO2-air layer, between 200 < z < 300 nm below the triskelion motif, as schematized in **Supplementary Figure 11**. Values of near-unity optical chirality can be found for the blue emitting region at λ = 449 nm (**a**). The green resonance at λ = 555 nm, as discussed in the main text, shows much weaker values of optical chirality density, hence the integrated value decreases rapidly and leads to a hindered CPE at this region (**b**). In the case of the third region of interest at λ = 667 nm, similar integrated values to the blue-region are obtained, therefore suggesting similar chiral strengths for both regions (**c**). Lastly, for the red emitting resonance centered at λ = 700 nm, the integrated values of excess of optical chirality reach values close to 20-fold in the flat high-index layer, and values close to 5-fold in the active region (**d**). This reinforces the experimental findings that the best performing wavelength for the chiral metasurface proposed is optimized for deep-red emitting materials.

Integrated optical chirality as a function of propagating distance

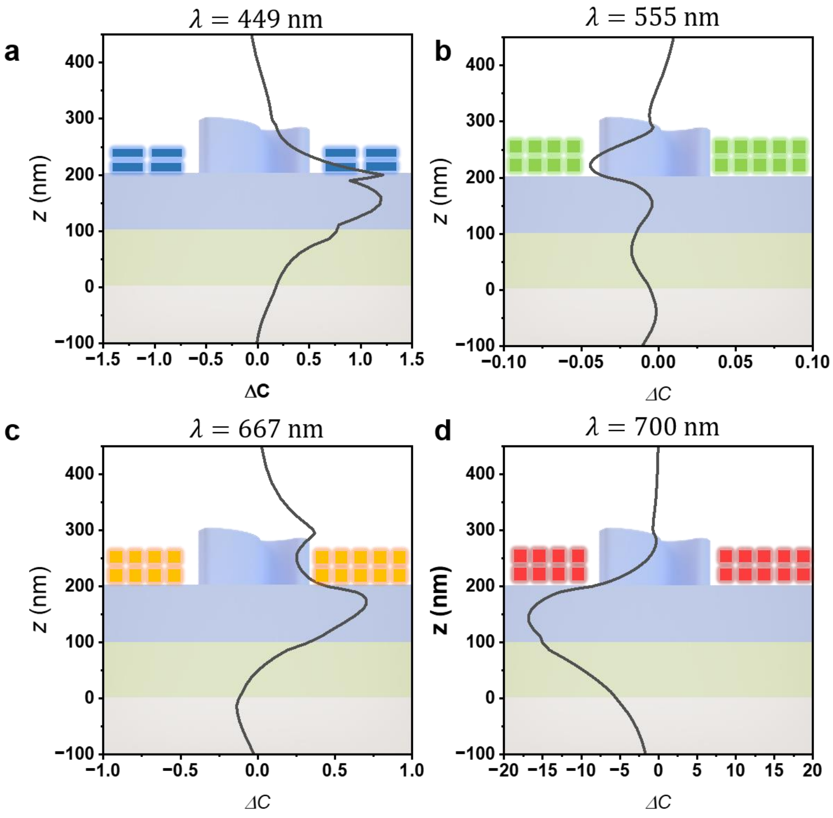

Supplementary Figure 11. Integrated in-plane optical chirality density as a function of propagation z-axis distance for **a**, 449 nm **b**, 555 nm **c**, 667 nm and **d**, 700 nm chiral resonance regions.

An equivalent study but for the electric field intensity is performed in **Supplementary Figure 12**. In agreement with the electric field intensity integrated along the z-axis presented in **Supplementary Figure 9**, we now integrate it in the xy-plane as a function of the propagation z-axis as:

$$I_{\mathrm{i}}(z) = \frac{\iint_{\mathrm{S}} E_{\mathrm{i}}^{2}(x,y,z) \cdot F(x,y) dxdy}{\iint_{\mathrm{S}} dxdy} \quad \textbf{(4)}$$

Where $E_{\mathrm{i}}^{2}(x,y,z)$ is the electric field intensity for the i polarization and $F(x,y)$ is a mask function to integrate only in the active region. Dashed and solid lines account for the electric field intensity integrated with and without applying the integration mask. At λ = 449 nm (**a**), we obtain a larger contribution for the RCP integrated intensity, thus resulting in an LCP emission based on reciprocity. For the broad resonance at λ = 555 nm (**b**), both contributions are similar, hence the expected dissymmetry at this wavelength is weaker. At λ = 667 nm (**c**), we obtain a higher contribution for the integrated LCP, resulting in a preferential RCP emission. Finally, at λ = 700 nm (**d**) the difference is very strong for the integrated LCP injection, therefore resulting in a strong RCP emission, as observed experimentally.

Integrated electric field intensity as a function of propagating distance

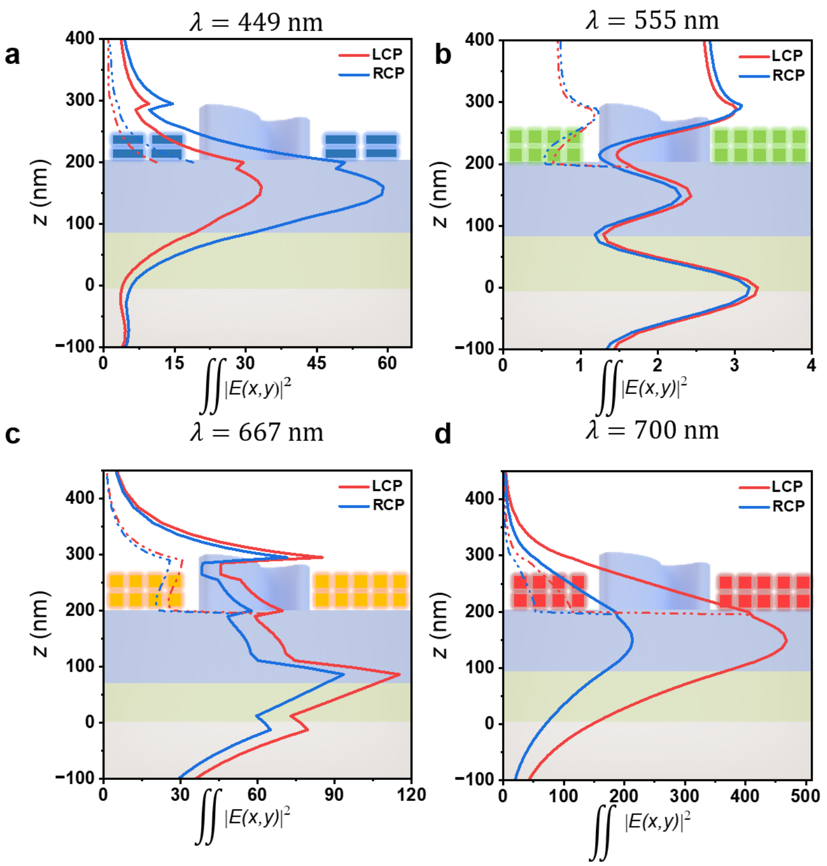

Supplementary Figure 12. Integrated electric field intensity for LCP (red) and RCP (blue) excitation as a function of propagation z-axis distance for **a**, 449 nm **b**, 555 nm **c**, 667 nm and **d**, 700 nm chiral resonance regions. Dashed lines correspond to the electric field intensity integrated into the air gaps applying the integration mask.

Supplementary note 2: Additional transmission and scanning electron microscopy characterization

We show the uniformity and the excellent quality of the chiral array metasurfaces fabricated using soft-nanoimprint lithography in **Supplementary Figure 13**, paving the way for large-area and scalable chiral nanophotonic fabrication methods for practical optoelectronic devices.

Low magnification scanning electron microscopy images

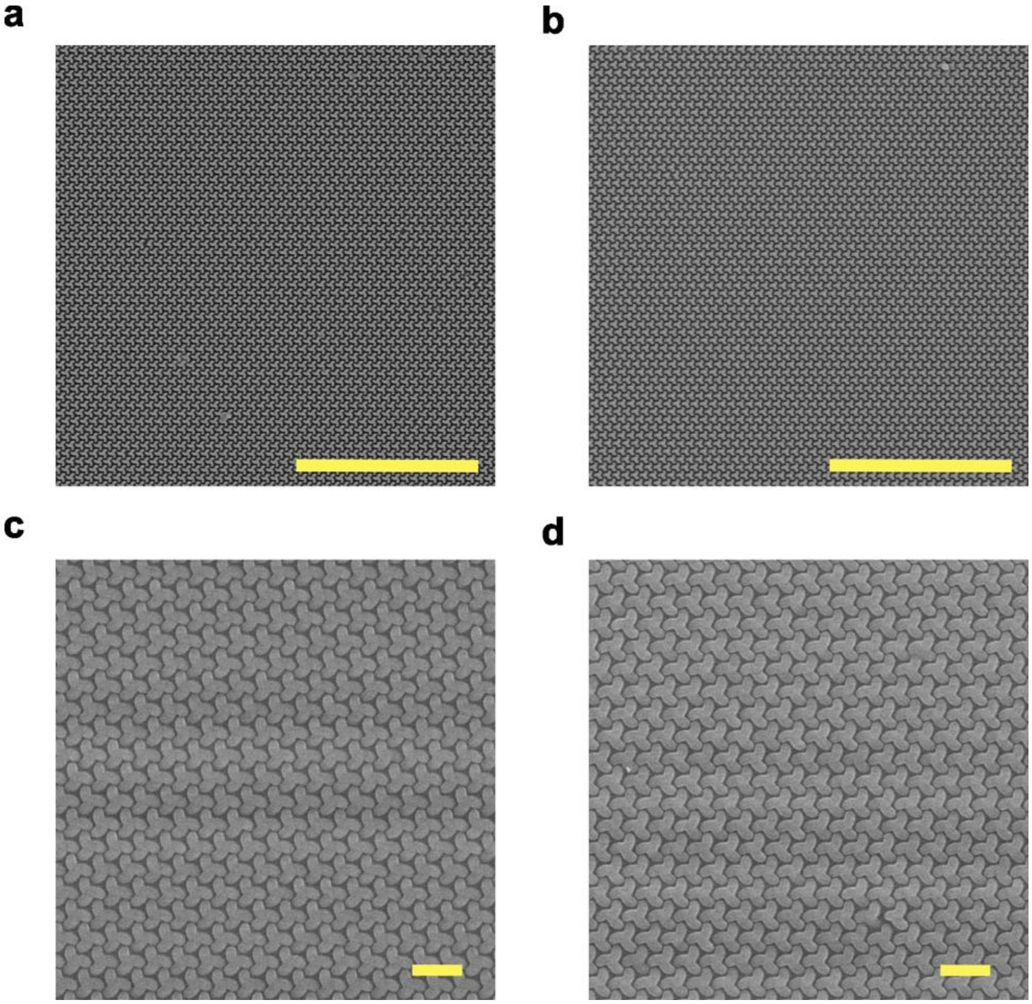

Supplementary Figure 13. Low (first row) and large (second row) magnification scanning electron microscopy images for uncoated (**a**,**c**) L- and (**b**,**d**) R-triskelion chiral metasurfaces. Scale bars are (**a,b**) 10 μm and (**c,d**) 1 μm.

Transmission electron microscopy characterization of the colloidal emitters can be found in **Supplementary Figure 14**. The excellent quality of the colloidal materials synthesized herein is clear from the presence of highly monodispersed nanoparticles in all images. Their colloidal stability and their excellent and easily tunable optical properties make these emitting materials excellent candidates to probe the nanophotonic platform.

Transmission electron microscopy images for colloidal emitters

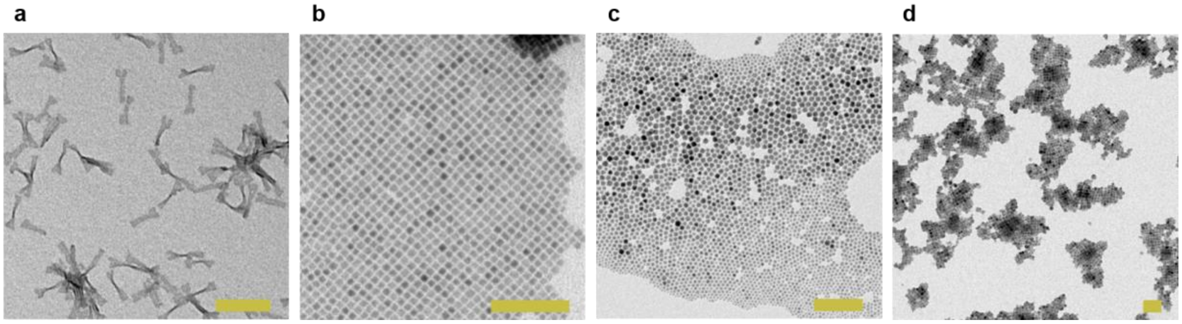

Supplementary Figure 14. Transmission electron microscopy images of **a,** CdSe/CdS core-crown nanoplatelets **b**, $CsPbBr_3$ perovskites nanocrystals **c**, CdSe/CdS core-shell quantum dots and **d**, $CsPbI_3$ perovskite nanocrystals. The scale bars indicate 100nm.

A high magnification SEM image for all the emitters after their deposition on our metasurfaces can be found in **Supplementary Figure *15*Supplementary Figure *16***. We observe that all the emitters assemble in the gaps between the triskelion motifs, where the optical chirality has been characterized in the previous section. Moreover, the colloidal emitters are uniformly distributed in most of the cases, thus reinforcing the deposition methods used herein to coat the nanophotonic metasurface.

High magnification scanning electron microscopy images (1)

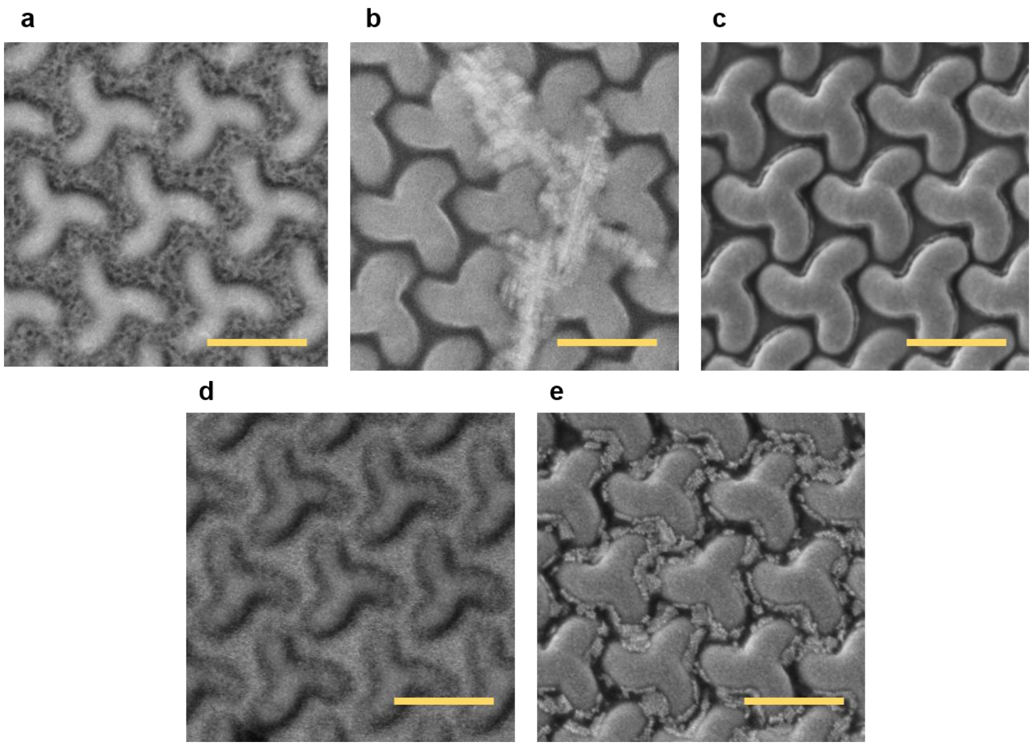

Supplementary Figure 15. High magnification SEM images of the emitting materials in a unit cell of the L-triskelion array for **a**, CdSe/CdS core-crown nanoplatelets **b**, $CsPbBr_3$ perovskite nanocrystals **c**, F8BT conjugated polymer **d**, CdSe/CdS core-shell quantum dots and **e**, $CsPbI_3$ perovskite nanocrystals. The scale bars indicate 500nm.

High magnification scanning electron microscopy images (2)

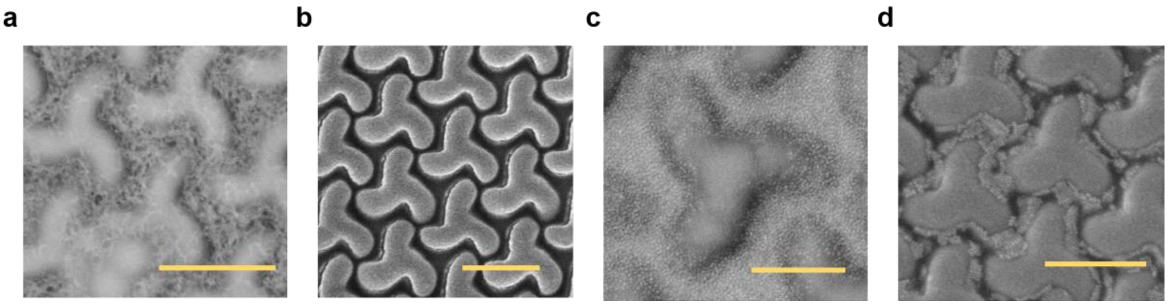

Supplementary Figure 16. High magnification of L-triskelion metasurface coated with **a** CdSe/CdS core-crown nanoplatelets **b**, F8BT conjugated polymer **c,** CdSe/CdS core-shell quantum dots and **d** $CsPbI_3$ perovskite nanocrystals. Scale bars are (**a, b, d**) 500 nm and (**c**) 250 nm.

We model the effect of the emitter material in two different approaches. First, we consider the deposition to be located at the walls of the triskelion motif, as observed in the SEM images. Secondly, a simpler approach where we model the emitters as a uniform thin layer

atop the TiO_2 flat coating, covering all the air gaps. We consider three types of materials, modeled as different refractive indices of n = 1.6, 1.8 and 2.2. Then, we compute the $\Delta T/T$ for the transmittance of LCP and RCP, presented in **Supplementary Figure 17**. The first thing to note in general terms is the redshifting of the resonances for increasing the refractive index, as expected. Secondly, is that the use of high-refractive index materials diminishes the resonance strength in the red part, even for small coating thicknesses. On the other hand, working with lower refractive index materials maintains the circular dichroism for thick layers, as observed in **Supplementary Figure 17a**,**b**. Even if the material's refractive index is higher, we can achieve a lower effective refractive index by decreasing the concentration. When the emitters are modeled as stuck to the walls, the response does not depend much on the thickness. On the other hand, when modeling the emitters as thin layers, the response can drastically change. For thin layers, we obtain similar responses as the uncoated case, with strong and sharp chiral resonances (**c**). However, when the thickness is above the triskelion motif, new guided-modes can now be excited, modifying completely the response of the system (**d**).

Modelling the effect of the emitters on top of the chiral metasurface

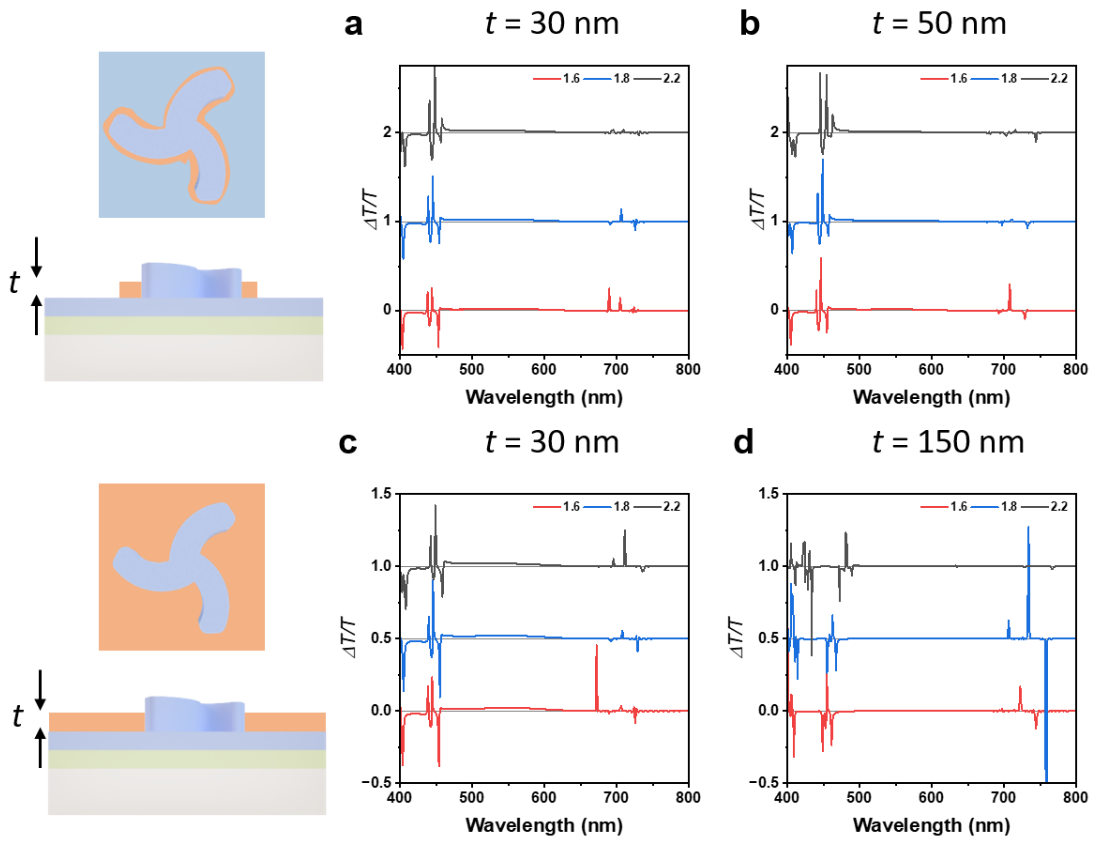

Supplementary Figure 17. Transmittance dissymmetry factor $\Delta T/T$ for emitters modeled as stick to the walls (top row) with thicknesses of **a**, 30 nm and **b**, 50 nm. Transmittance dissymmetry factor $\Delta T/T$ for emitters modeled as a thin layer (bottom row) for thicknesses of **c**, 30 nm and **d**, 150 nm.

Supplementary note 3: Chiral photoluminescence additional information

3.1 Circularly polarized photoluminescence from unpatterned films

As a control measurement, we measure the circularly polarized emission exciting in the unpatterned area, where the emitters are disposed in a flat layer of TiO_2. As no chiral photonic environment is near the emitters, there is no preferential emission when filtering the signal for left and right circularly polarized light, as shown in **Supplementary Figure 18**. Note that, for F8BT organic polymer emission (**c**), no enhanced photoluminescence peaks are observed at the emission tails as for the case of emission coming from patterned areas shown in the main text. The same argument is valid for CdSe/CdS quantum dots (**d**) and red $CsPbI_3$ perovskites (**e**).

Circularly polarized emission from unpatterned areas

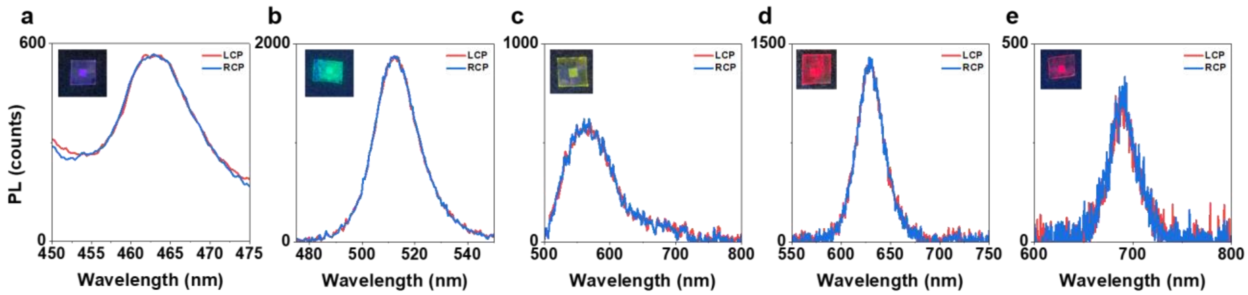

Supplementary Figure 18. Circularly polarized emission from unpatterned areas for **a**, CdSe/CdS core-crowned nanoplatelets **b**, $CsPbBr_3$ nanocrystals **c**, F8BT conjugated polymer **d**, CdSe/CdS core-shell quantum dots **e**, $CsPbI_3$. Inset: Macroscopic image of the metasurface coated with different emitters

3.2 Unraveling the effect of parameters in the CPE

The dissymmetry factor presented in **Fig. 1c** corresponds to one of the fabricated chiral metasurfaces. However, various parameters during the nanofabrication process can affect the final optical response, such as the deposited TiO_2 thickness, the height of the triskelia, or the homogeneity of the sample in a sufficiently large area. These parameters can affect the strength of the chiral resonances discussed, but the overall response of the system remains equivalent. This is shown in **Supplementary Figure 19a**, where the transmittance dissymmetry factor for the metasurfaces used for each emitter is shown. The overall response is in good agreement with the one reported in **Fig. 1c**. However, and more visible in the red part of the spectrum, the discrepancies during the nanofabrication process may induce spectral shifting or more intricate behavior such as change of signs in the dichroism. This in turn results in a complicated behavior for the CPE. As an example, we show the transmittance and emission dissymmetry factors for the broadband emitter L-triskelion coated with F8BT in **Supplementary Figure 19b**. Even though the measured g_{lum} does not fully agree with the

transmittance presented in **Fig. 1c**, it shows a strong correlation when comparing it to the measured $\Delta T/T$ on the same metasurface. These changes, as stated, can be linked to variations during the nanofabrication process, as we will discuss further in this section.

Comparing the dissymmetric transmittance and emission for the different metasurfaces

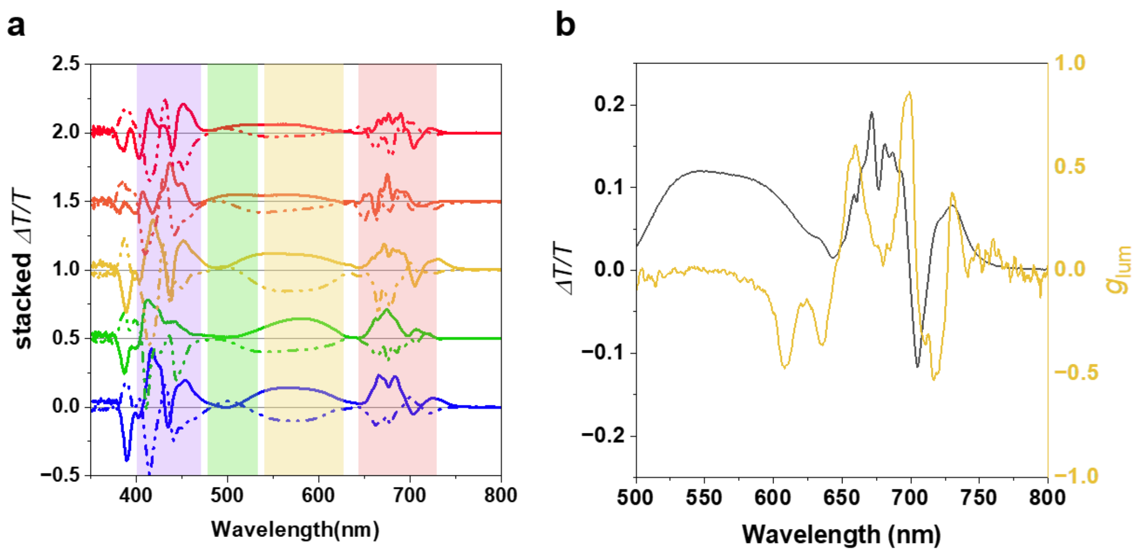

Supplementary Figure 19. a, Experimental transmittance dissymmetry factor $\Delta T/T$ of L- (solid line) and R-triskelion (dashed line) for, from bottom to top, CdSe/CdS core-crowned nanoplatelets **(**blue**)**, $CsPbBr_3$ nanocrystals(green), F8BT conjugated polymer (yellow) CdSe/CdS core-shell quantum dots (orange) and $CsPbI_3$ (red). **b**, Transmittance $\Delta T/T$ (solid dark) and emission g_{lum} (solid yellow) dissymmetry factors for L-triskelion coated with F8BT conjugated polymer.

We investigate the effect of different parameters in the CPE for the emitters used herein. Therefore, we will use a Lorentzian lineshape as a weighting function to model the PL spectra of the emitters at various wavelengths (**Supplementary Figure 20**). We consider the peak emission of the colloidal emitters of CdSe/CdS core-crowned nanoplatelets **(**blue**)**, $CsPbBr_3$ nanocrystals (green), CdSe/CdS core-shell quantum dots (orange), and $CsPbI_3$ perovskites(red), centered at 460 nm, 510 nm, 630 nm and 680 nm, respectively.

Weighting Lorentzian functions to account for photoluminescence processes

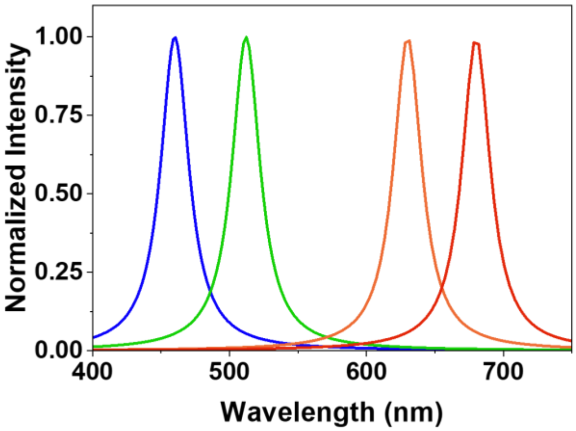

Supplementary Figure 20. Normalized intensity of the Lorentzian profile used as weighting function for photoluminescence simulations centered at, from left to right, 460 nm (blue), 510 nm (green), 630 nm (orange) and 680 nm (red).

In order to model the photoluminescence process within the array, we model the metasurface as a finite array of 18x18 unit cells and place a monitor at the glass substrate to compute the far-field emission at this side. From the monitor, we compute the far-field polarization components of Es and Ep and from them obtain the circular components as[4]:

$$I_{\mathrm{LCP}}(\mathbf{u}_{\mathrm{x}}, \mathbf{u}_{\mathrm{y}}) = \frac{1}{2}\left(\mathbf{E}_{\mathrm{p}}\mathbf{E}_{\mathrm{p}}^{*} + j\mathbf{E}_{\mathrm{p}}^{*}\mathbf{E}_{\mathrm{s}} - j\mathbf{E}_{\mathrm{p}}\mathbf{E}_{\mathrm{s}}^{*} + \mathbf{E}_{\mathrm{s}}\mathbf{E}_{\mathrm{s}}^{*}\right) \quad (5)$$

$$I_{\mathrm{RCP}}(\mathbf{u}_{\mathrm{x}}, \mathbf{u}_{\mathrm{y}}) = \frac{1}{2}\left(\mathbf{E}_{\mathrm{p}}\mathbf{E}_{\mathbf{p}}^{*} - j\mathbf{E}_{\mathrm{p}}^{*}\mathbf{E}_{\mathrm{s}} + j\mathbf{E}_{\mathrm{p}}\mathbf{E}_{\mathrm{s}}^{*} + \mathbf{E}_{\mathrm{s}}\mathbf{E}_{\mathrm{s}}^{*}\right) \quad (6)$$

Where $\mathbf{u}_x$ and $\mathbf{u}_y$ are the unit vectors for the propagating radiation. We then can calculate the portion of each polarization that would reach the detector by defining our numerical aperture (NA). From the total amount of intensity, we can extract the proportion for each polarization for each wavelength and then weight it with the Lorentzian functions previously discussed. Consequently, we can measure the theoretical amount of energy for each polarization that would reach the detector.

Supplementary Figure 21 summarizes the results obtained for the CPE measured when varying various parameters. First, **a** and **b** consider the variation of the SU8 triskelion height and the coating of TiO_2, respectively. Then, **c, d**, and **e** consider the CPE dependence for different positions of the dipole, orientation, and collection NA. The simulation for varying positions (**c**) is averaged for the three possible orientations of the dipole. On the other hand, the simulation for varying orientation (**d**) is averaged at different positions within the unit cell. The simulation for varying NA (**e**) is averaged in four different positions and the three orientations to account for all the possible emission variations within a real emission process. Except for the latter, all the simulations are considered in the normal direction and we assume an NA of 3 degrees.

These simulations try to illustrate that the photoluminescence processes are very sensitive to the local variations of the geometrical parameters. Specifically, we can see that variations in both the triskelion height (**a**) and the coating thicknesses (**b**) can switch the preferential emission signs at the peak wavelengths. Therefore, variations within the chiral metasurface may disturb our preferential CPE. For the simulation parameters used for the optimized geometry (h = 160 nm, t = 95 nm) we then calculate the CPE dependence for various positions of the dipole within the unit cell (**c**). We observe that, the emitters experience a strong emission dissymmetry when placed in the hotspots of the triskelia array (red cross). In other positions, the emission dissymmetry is lowered, therefore obtaining a decrease in the differential emission. Considering now the orientation of the excited dipole within the unit cell, we also observe differences in the far-field emission pattern (**d**). We observe that stronger enhancements are obtained for z-dipole oriented. However, this out-of-plane orientation cannot be excited at normal incidence and does not contribute much to the emission process. The orientations for x and y dipoles are in good agreement with the polarizations obtained experimentally. Finally, the dependence of the CPE on the collection NA is studied (**e**). We consider 3°, 10°, and 45° as different NA for the measured CPE. We observe that, for low NA at normal direction, we obtain CPE for blue, orange and red, in good agreement with the experimental results. In neither of the cases, green emission shows differential CPE. However, when increasing the collection angle, the obtained differential CPE is diminished for all wavelengths, therefore reinforcing our hypothesis of out-coupling efficiency at off-normal angles for one of the polarizations.

Relevant parameters affecting circularly polarized emission

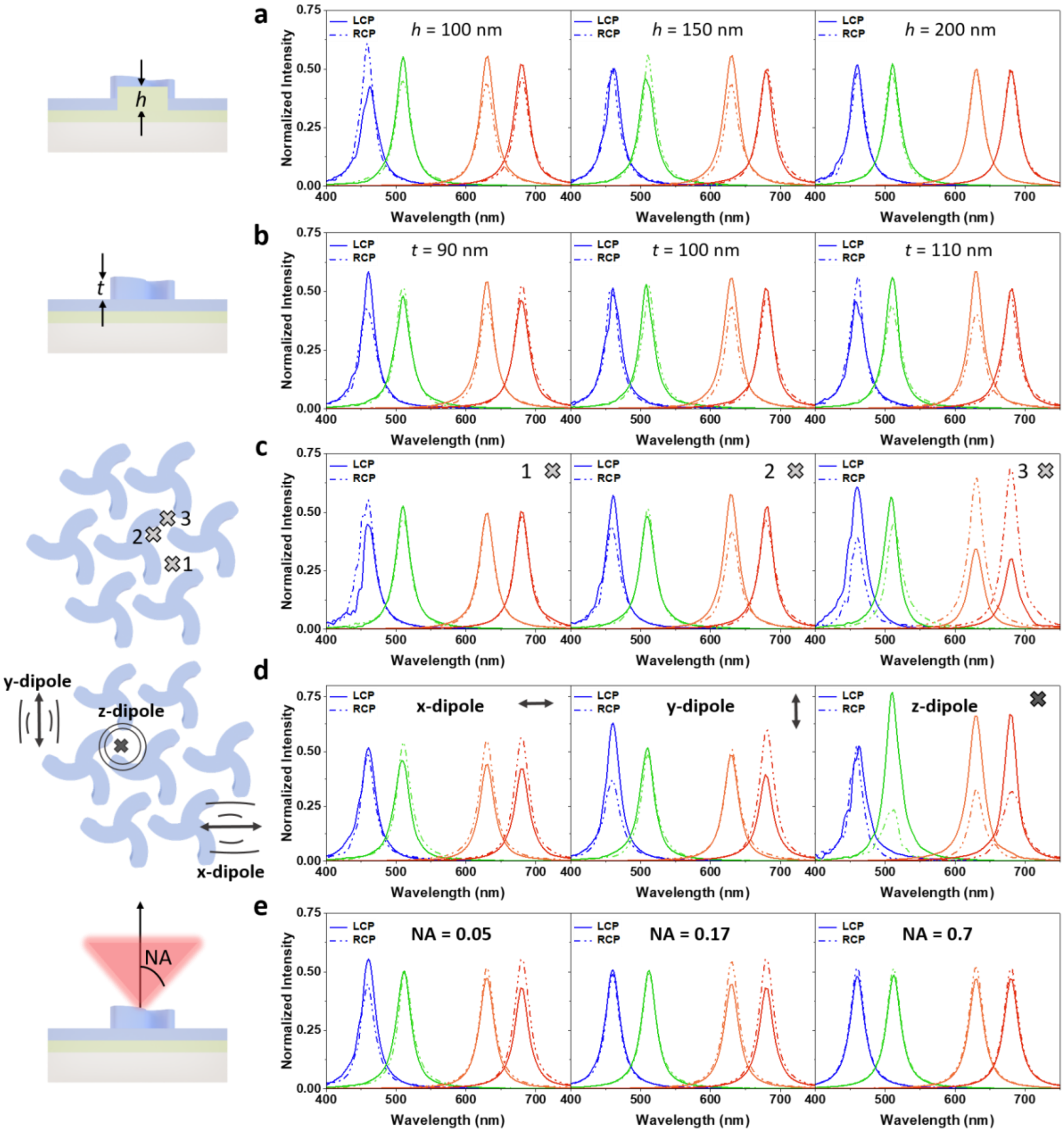

Supplementary Figure 21. Simulation of photoluminescence emission for LCP (solid line) and RCP (dashed line) when varying **a**, SU8 triskelion height **b**, TiO_2 coating thickness **c**, the position of the emitting dipole **d**, dipole orientation and **e**, numerical aperture of the collection system.

All the discussions presented in this work have neglected angular dependence. However, it is known that these metasurfaces can respond differently for each polarization as a function of the incident angle[3,5]. Even though it is beyond the scope of this work, as it is focused on the emission near the Γ-point, we experimentally characterize the angular response of the metasurfaces under LCP and RCP waves (**Supplementary Figure 22**). Moreover, as the

array is composed in a hexagonal lattice, we inspect both orientations around the Γ-point, to the ΓX (first row) and ΓM (second row). Indeed, the angular response of the chiral metasurface does show differences for opposite polarizations, in-coupling diffractive modes differently for LCP (**a,d**) and RCP (**b,e**). The diffractive modes are coupled in both blue and red resonances, reinforcing the idea of lattice modes, whereas the broad green resonance remains unmodified for larger incident angles. This is clearly seen when inspecting the angular transmittance dissymmetry factor for both orientations (**c,f**) where the red and blue colored lines indicate enhanced in-coupling for LCP and RCP polarizations into diffractive modes, respectively. This, in turn, could be used for further polarized angular emission, as previously reported elsewhere[3,5,6].

Experimental angular transmittance and dissymmetric factor

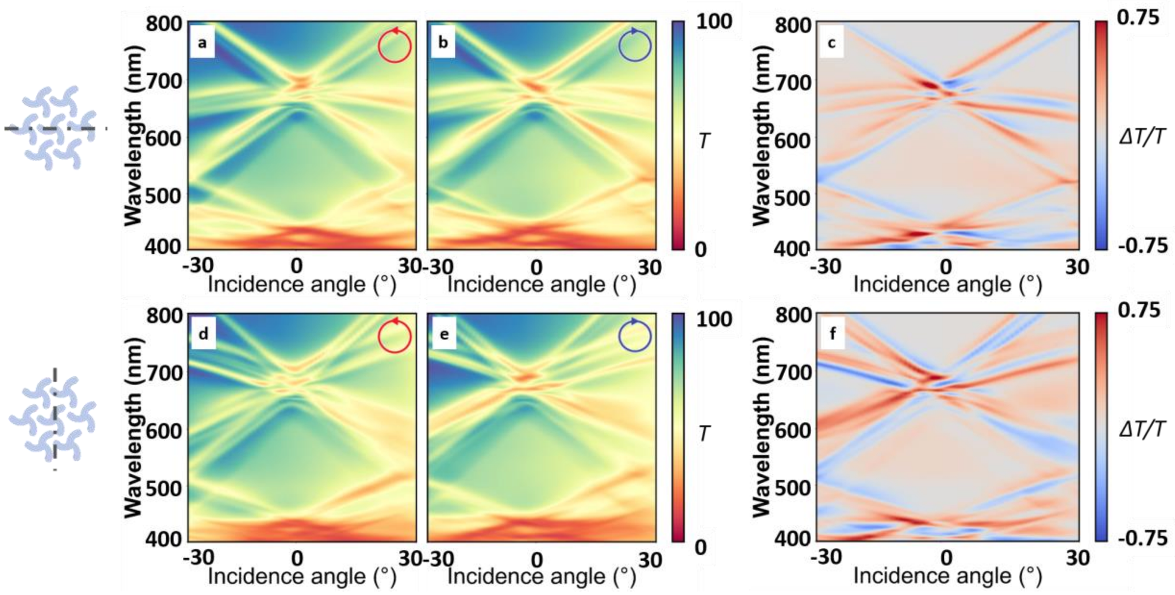

Supplementary Figure 22. Experimental angular transmittance for LCP (red) and RCP (blue) at (**a,b**) 0° and (**d,e**) 90° azimuthal angles. The corresponding differential transmission in each case is shown in (**c**) 0° and (**f**) 90°.

We investigate now the dependence on the CPE as a function of the emission angle summarized in **Supplementary Figure 23**. For the same colloidal stability reasons discussed in the main text, we study this emission property for the CdSe/CdS quantum dots. We analyze both L- and R-triskelion metasurfaces at various emission angles. It is worth noting that there are several enhanced emission peaks corresponding to various diffraction conditions, therefore, it is involved to follow the modes at different angles. For this reason, we have added a dark dashed line as a guide for the eye to focus on a resonant mode for each enantiomer. As discussed previously in this section, discrepancies in the local geometrical parameters may perturb the resonant wavelength, as we observe in the vast amount of diffraction peaks. However, if we follow the mode selected with the guide for the eye, we can see that the polarization handedness is maintained at various angles, and it blueshifts for increasing angle, going from 652 nm at normal incidence to 610 nm. Therefore, this opens

up a new way for tuning both the polarization handedness and the emission wavelength by tuning the diffraction condition to the proper emission angle[5]. By doing so, we can obtain larger emission dissymmetric g_{lum} values. For the L-triskelion, we can tune from 0.4 at 10°, -0.53 at 15°, and 0.88 at 20° for 639 nm, 619 nm and 606 nm, respectively. As for the R-triskelion, we can tune from -0.8 at 10°, -1.32 at 15° and -1.35 at 20° for the same resonant wavelengths.

Experimental angular circularly polarized emission

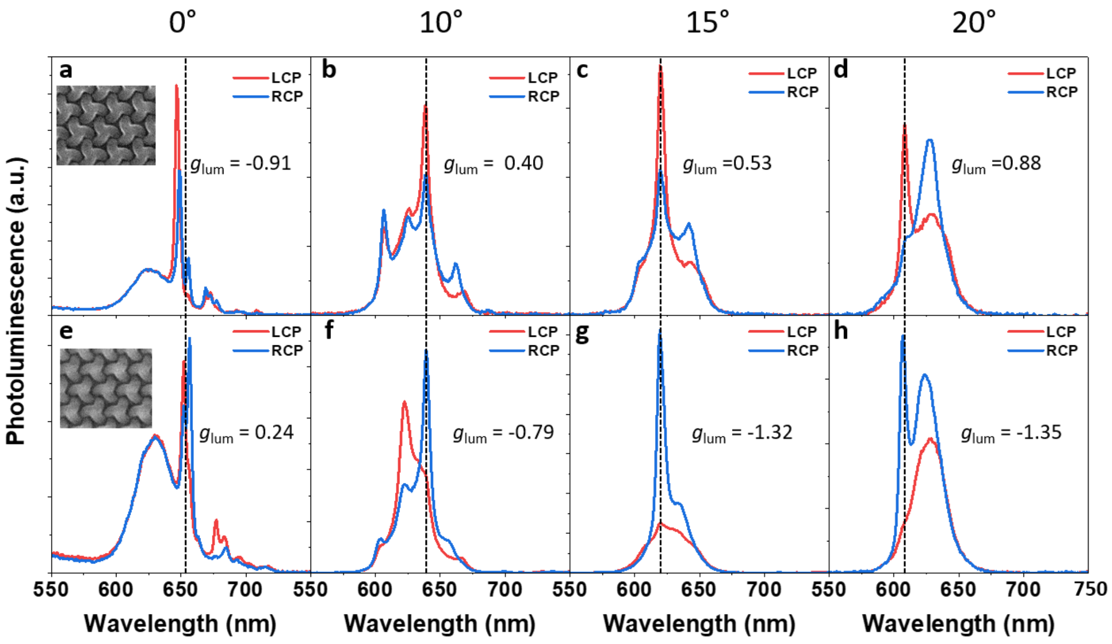

Supplementary Figure 23. Angular CPE for L- (top row) and R-triskelion (bottom row) coated with CdSe/CdS quantum dots at 0° (**a, e**) 10° (**b, f**), 15° (**c, g**) and 20° (**d, h**). The dashed dark line is a guide for the eye to follow the diffractive mode outcoupled. Inset: SEM image of L- (**a**) and R-triskelion (**e**) metasurfaces.

Finally, we measure the CPE in steps of 2° and collect from -30° to +30° the emission of both LCP and RCP contributions. From these measurements, we compute the emission dissymmetric factor g_{lum} as a function of the emission angle, shown in **Supplementary Figure 24**. As a guide for the eye, we compute the angular dependence of the diffractive resonances present in a hexagonal lattice, shown as dark dashed lines overlapping the experimental data. We calculate them as:

$$\mathbf{k}_{//}{}^{\mathrm{out}} = \mathbf{k}_{//}{}^{\mathrm{in}} + \mathbf{G}_{\mathrm{m,n}} = \frac{2\pi}{\lambda}(\cos\varphi\sin\theta, \sin\varphi\sin\theta) + \frac{2\pi}{\Lambda}(m\mathbf{b_1} + n\mathbf{b_2}) \quad (7)$$

$$\mathbf{b_1} = \frac{1}{\sqrt{3}}\left(\sqrt{3}\mathbf{x} - \mathbf{y}\right); \mathbf{b_2} = \frac{2}{\sqrt{3}}\mathbf{y} \quad (8)$$

Where φ and θ are the azimuthal and polar angle of the metasurface for the incident light, and (m,n) are integers representing the diffraction order associated with the reciprocal lattice vector $\mathbf{G}_{\mathrm{m,n}}$. The represented diffractive lines are associated with the incident parameters corresponding to normal incidence $\theta = 0º$ and with a small azimuthal of $\varphi = 4º$ to match the experimental data. The diffracted wave vector is associated with an effective medium between the glass (n = 1.49) and the SU8 (n=1.6) refractive indices of $n_{\mathrm{eff}} = 1.54$.

It is clearly seen that the CPE follows the diffraction lines of the array (marked as dashed dark lines), indicated as either red (for LCP) or blue lines (for RCP) and, consequently, we are benefiting from the diffractive radiative channel for the preferential out-coupling efficiency. Nonetheless, we observe that the emission at negative and positive angles is equivalently mirror symmetric. Indeed, the angular response depends on various parameters, such as the orientation of the sample or the excitation polarization plane. Therefore, it requires a much detailed study which is beyond the scope of this work. Future studies will address in detail the angular dependence of the structure presented herein.

Experimental dissymmetric angular circularly polarized emission

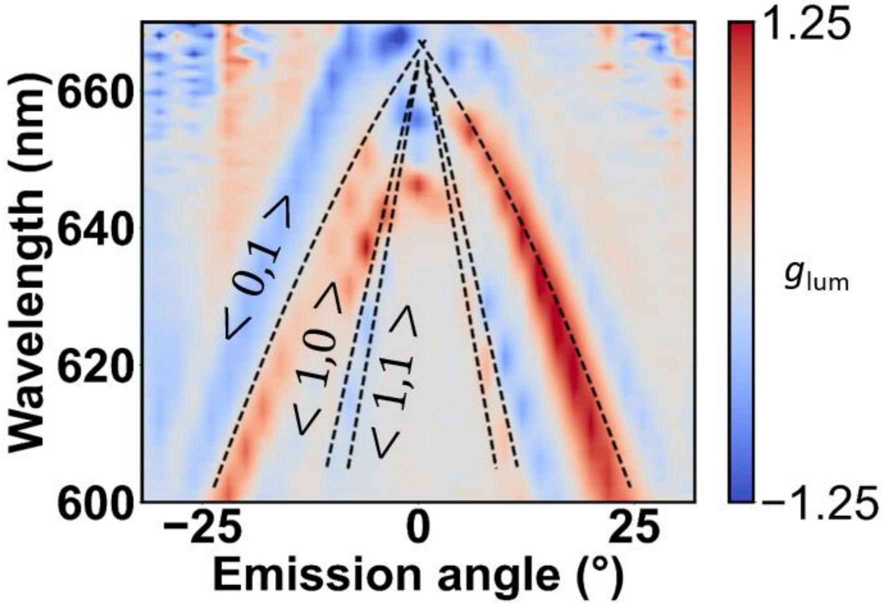

Supplementary Figure 24. Dissymmetry emission factor g_{lum} for L-triskelion coated with CdSe/CdS quantum dots.

As a control measurement, we also measured the angular response of the emission from the unpatterned substrate (**Supplementary Figure 25**). As expected, no diffraction conditions are met for the unpatterned case, and therefore, the CPE for both helicities is equivalent.

Experimental dissymmetric angular circularly polarized emission from unpatterned areas

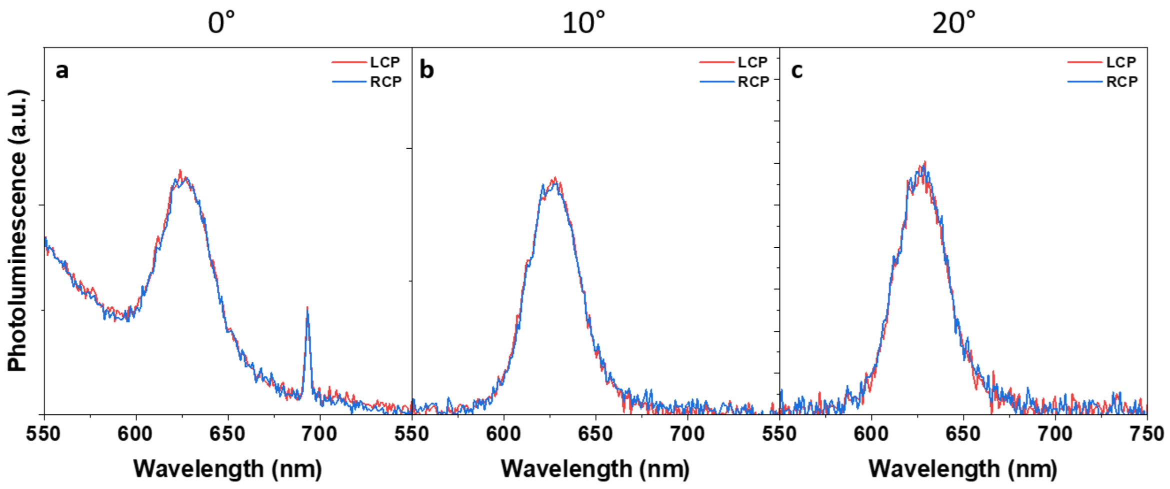

Supplementary Figure 25. Angular CPE for unpatterned emission for **a**, 0° **b**,10° and **c**, 20°

3.3 Unpolarized LED excitation photoluminescence

As discussed in the main text, an LED unpolarized excitation source is used to prevent any photoselection process during the absorption of the exciting radiation. Lasers are inherently polarized radiation due to their generation mechanism and may transfer their polarization orientation to the dipolar emission of molecules or nanoemitting materials. It is convenient to remove this source of uncertainty when measuring circularly polarized emission, as the preferred orientation may lead to artifacts[7]. However, we observe similar circularly polarized emission spectra when compared to the laser excitation sources, as shown in **Supplementary Figure 26Supplementary *Figure 27*** for L- and R-triskelion, respectively. The chiral metasurfaces display preferential emission of a given helicity even at low power densities, thus suggesting that the polarization induction occurs due to the triskelion scattering acting as a chiral antenna.

Experimental circularly polarized emission of L-triskelion under LED excitation

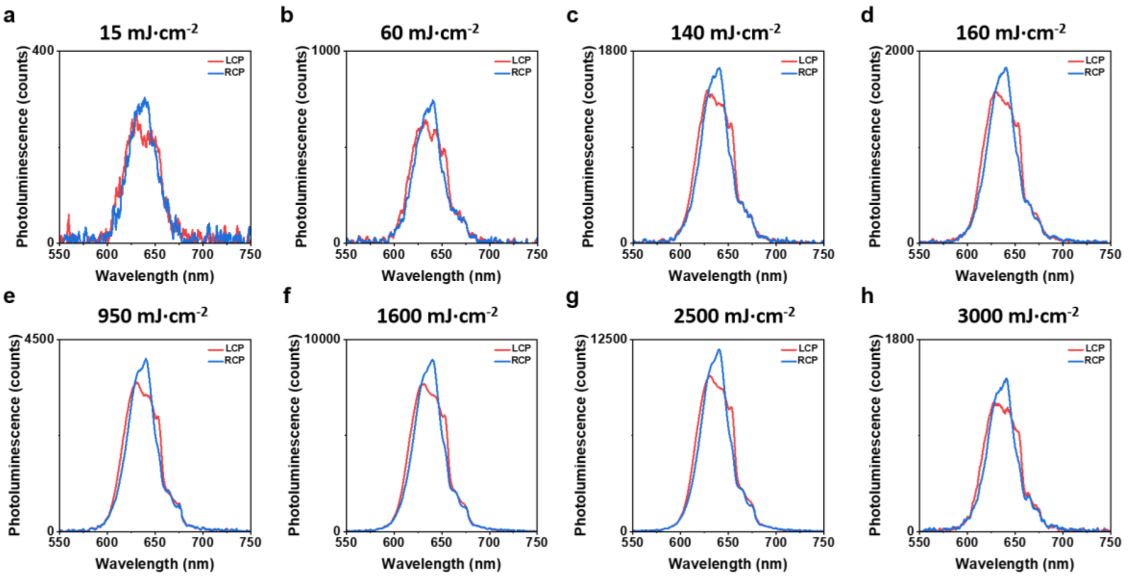

Supplementary Figure 26. Circularly polarized emission for CdSe/CdS core-shell quantum dots in L-triskelion chiral metasurface for different LED excitation power densities.

Experimental circularly polarized emission of R-triskelion under LED excitation

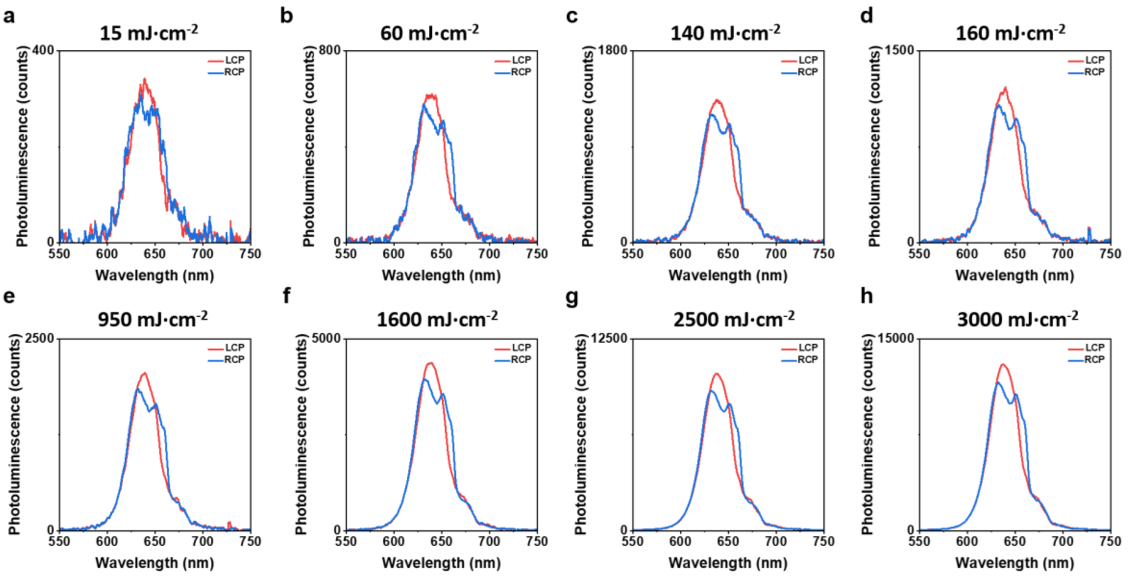

Supplementary Figure 27. Circularly polarized emission for CdSe/CdS core-shell quantum dots in R-triskelion chiral metasurface for different LED excitation power densities.

3.4 Additional information on time-resolved chiral photoluminescence

The rate of emission of a luminescent material depends on the local environment's optical properties. The coupling of the emitting material to microcavities or inhomogeneous media leads to a modification of the photon emission rates compared to those in vacuum, known as the Purcell effect[8]. A change in the lifetime decay rates indicates an effective coupling of the emitter to the resonant microcavity. However, no differences in the decay rate are observed when comparing the normalized lifetime of the circularly polarized light emission from the patterned and unpatterned substrates, specifically right-circularly polarized emission (**Supplementary Figure 28**). This suggests that the PL dynamics of the emitting material are not affected by the photonic architecture.

Experimental time-resolved circularly polarized decay comparing metasurface and unpatterned areas

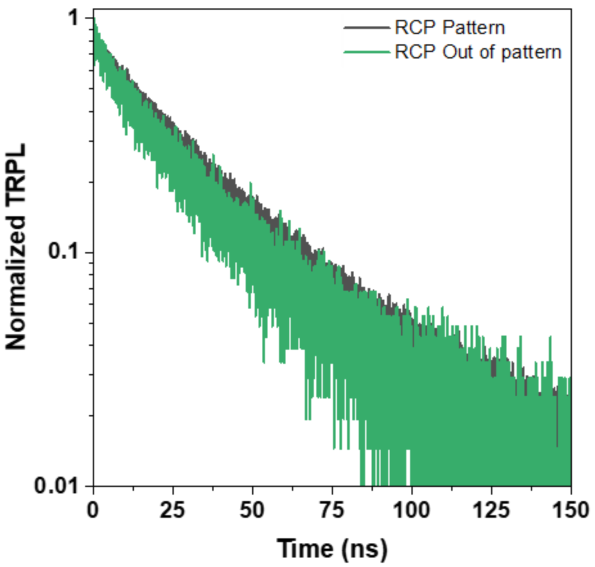

Supplementary Figure 28. Normalized right-circularly polarized emission decay curves for emission from patterned and unpatterned sample regions

As discussed in the main text, the differential emission intensities for both circularly polarized emissions are not transferred to the decay emission rates for both polarizations, showing an identical PL lifetime when normalized. However, when the number of photons for both polarizations is measured during the same time window, larger values are observed for the preferential emitting handedness. When inspecting the time-resolved photoluminescence of the L-triskelion coated with CdSe/CdS quantum dots at a wavelength where the chiral dissymmetry is maximum (red-colored shaded area in **Supplementary Figure 29a**), a higher amount of RCP emitted photons reach the detector, shown in **Supplementary Figure 29c**, as in the case of the steady-state analysis. As expected, an

opposite behavior is observed for the R-triskelion metasurface, where a preferential left-circularly polarized light emitted at 640nm (**Supplementary Figure 29b)** accumulates a larger number of photons in the dynamic measurements, shown in **Supplementary Figure 29d**.

Experimental comparison of steady-state and time-resolved circularly polarized emission

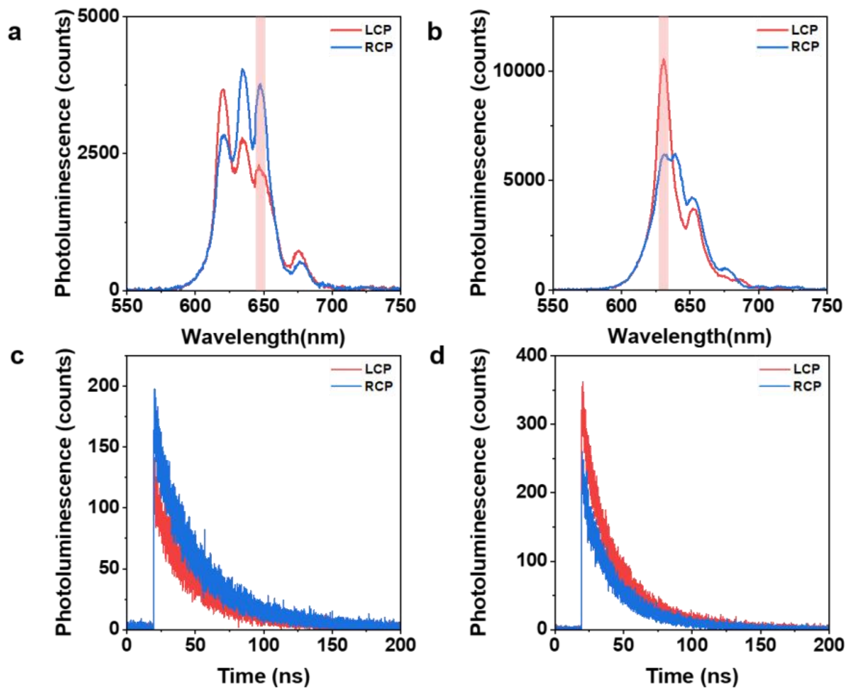

Supplementary Figure 29. Circularly polarized emission and time-resolved chiral photoluminescence of CdSe/CdS core-shell quantum dots for L- (**a**,**c**) and R-triskelion (**b**,**d**). The red-shaded area indicates the working spectral window of the monochromator for the time-resolved chiral photoluminescence measured.

We performed a set of independent measurements for the emission of the L-triskelion metasurface coated with CdSe/CdS QDs filtering both circularly polarized emissions to rely on statistical differences, if present. At the emission wavelength of 648nm, we performed 8 different measurements for each polarization with a fixed integration time. We adjusted each of these decay curves with a double exponential model, accounting for both surface (fast) and core excitonic recombinations[9]. The fitting process was performed fixing a time window for the convergence of the fitting algorithm to guarantee that the boundary conditions were equivalent for all the measurements and polarizations. The number of photons accounting for the decay of the exciton in the CdSe/CdS QDs is modeled as:

$$y = y_0 + A_1 e^{-t/\tau_1} + A_2 e^{-t/\tau_2} \qquad (9)$$

Where y is the number of photons measured, y_0 accounts for the background noise, and A_i and τ_i describe the amplitude and the decay time for each decay process.

A mean value for the lifetime is calculated, taking the relative amplitude and decay rates for both processes as

$$\langle \tau_\mathrm{i} \rangle = \frac{\sum_\mathrm{i} A_\mathrm{i} \tau_\mathrm{i}^2}{\sum_\mathrm{i} A_\mathrm{i} \tau_\mathrm{i}} \qquad (10)$$

The deviations for the calculated mean value are retrieved using uncertainty propagation formulas and determine whether the differences for the mean value of each polarization reside within the experimental error. The calculated values for LCP and RCP mean lifetimes are 25.5 ± 1.2 and 24.9 ± 1.2 ns, respectively. Hence, both values are within the same uncertainty interval and no lifetime differences are observed.

**LCP**	**y_0**	**δy_0**	**A_1**	**δA_1**	**A_2**	**δA_2**	**τ_1 (ns)**	**$\delta\tau_1$**	**τ_2 (ns)**	**$\delta\tau_2$**	**R^2**
**1**	0.012	0.0013	1.34	0.19	1.09	0.07	9.8	1.19	32.11	0.94	0.971
**2**	0.011	0.0002	1.14	0.09	1.04	0.11	12.0	1.71	33.58	1.55	0.971
**3**	0.011	0.0014	1.93	0.37	1.17	0.06	8.7	1.01	32.48	0.82	0.969
**4**	0.012	0.0011	2.05	0.60	1.18	0.04	7.6	1.10	31.31	0.63	0.969
**5**	0.012	0.0013	1.60	0.24	1.19	0.06	9.3	1.06	32.36	0.86	0.971
**6**	0.011	0.0011	1.39	0.24	1.13	0.06	9.1	1.13	31.71	0.77	0.972
**7**	0.012	0.0010	2.01	0.38	1.22	0.05	8.3	0.88	31.75	0.67	0.972
**8**	0.012	0.0013	1.73	0.28	1.20	0.06	9.0	1.00	32.26	0.80	0.970
**Mean**	0.012	0.0011	1.65	0.30	1.15	0.06	9.2	1.13	32.19	0.88	0.971

$$\langle \tau_{\mathrm{LCP}} \rangle = (25.5 \pm 1.2)\ \mathrm{ns}$$

Supplementary Table 1. Fitting parameters obtained of L-triskelion coated with CdSe/CdS quantum dots for the statistical retrieval of mean lifetime for left-circularly polarized light emission at 648 nm.

RCP	y_0	δy_0	A_1	δA_1	A_2	δA_2	τ_1 (ns)	$\delta\tau_1$	τ_2 (ns)	$\delta\tau_2$	R^2
**1**	0.014	0.0010	2.16	0.44	1.28	0.04	7.7	0.79	31.28	0.54	0.977
**2**	0.012	0.0011	1.62	0.18	1.15	0.05	9.6	0.89	33.01	0.79	0.978
**3**	0.013	0.0010	2.23	0.48	1.29	0.03	7.5	0.78	31.40	0.51	0.977
**4**	0.013	0.0010	2.57	0.52	1.26	0.04	7.4	0.68	31.82	0.52	0.977
**5**	0.012	0.0009	1.60	0.30	1.22	0.04	8.4	0.92	31.68	0.58	0.978
**6**	0.012	0.0010	1.62	0.27	1.20	0.05	8.7	0.94	32.14	0.65	0.976
**7**	0.013	0.0010	2.33	0.47	1.26	0.04	7.6	0.72	31.94	0.52	0.977
**8**	0.012	0.0010	1.99	0.29	1.20	0.04	8.6	0.77	32.56	0.64	0.977
**Mean**	0.012	0.0010	2.01	0.37	1.23	0.04	8.2	0.81	31.98	0.59	0.977

$$\langle \tau_{RCP} \rangle = (24.9 \pm 1.2)\ \text{ns}$$

Supplementary Table 2. Fitting parameters obtained of L-triskelion coated with CdSe/CdS core-shell quantum dots for the statistical retrieval of mean lifetime for right-circularly polarized light emission at 648 nm.

As expected, neither the flat TiO_2 unpatterned layer affects the lifetime decay rates of both circularly polarized emissions for any of the emitters, as shown in **Supplementary Figure 30**.

Experimental time-resolved circularly polarized decay of unpatterned areas

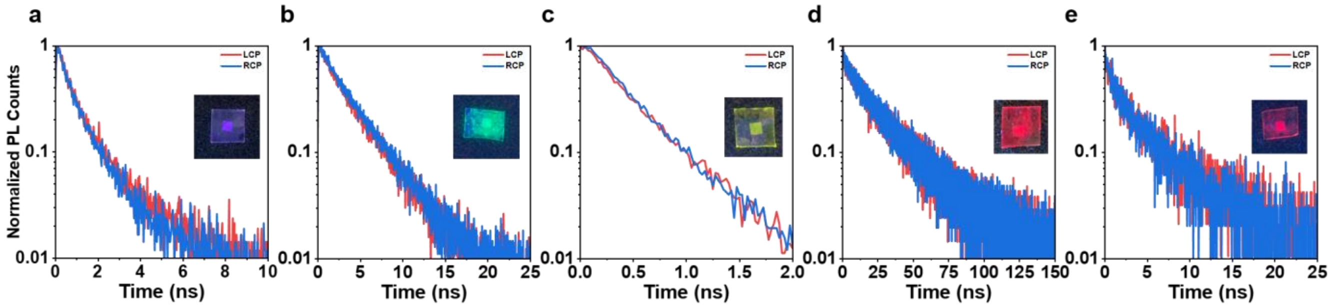

Supplementary Figure 30. Time-resolved chiral photoluminescence of the unpatterned emission for **a**, CdSe/CdS core-crown nanoplatelets **b**, $CsPbBr_3$ nanocrystals **c**, F8BT organic polymer **d**, CdSe/CdS core-shell quantum dots **e**, $CsPbI_3$. Inset: Macroscopic image of the metasurface coated with different emitters

Dynamic photoluminescence is also explored for the resonant wavelengths where the steady-state chiral photoluminescence showed a preferential emission for the rest of the nanomaterials used herein. Nevertheless, there were no observable differences in the decay rates of each polarization for any of the emitters, as shown in **Supplementary Figure 31**.

Experimental time-resolved circularly polarized decay of the different emitters

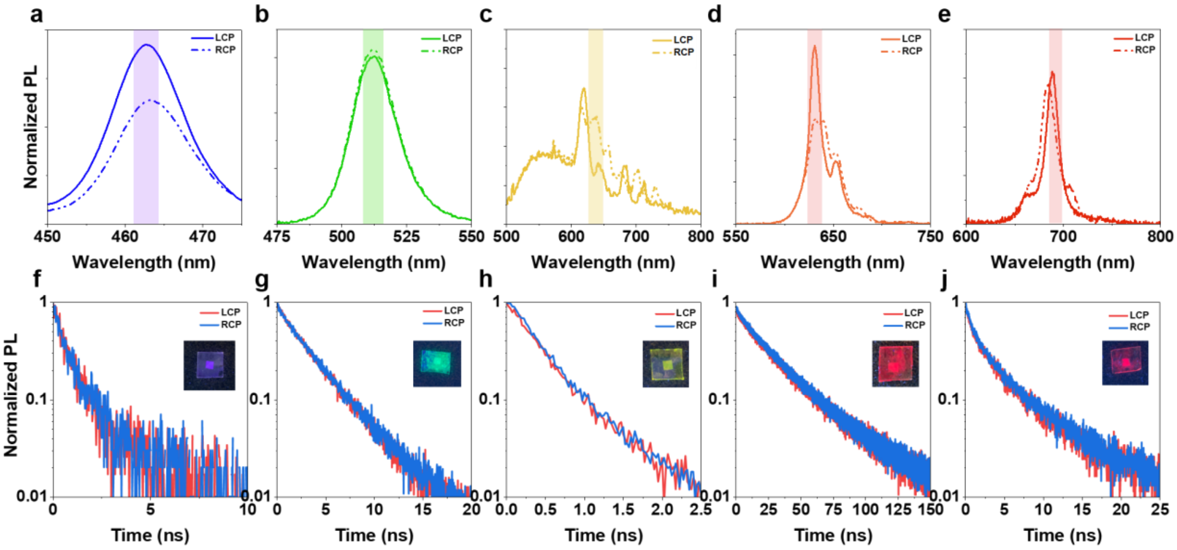

Supplementary Figure 31. Time-resolved chiral photoluminescence of L-triskelion array in the patterned emission for **a**, CdSe/CdS core-crown nanoplatelets **b**, $CsPbBr_3$ nanocrystals **c**, F8BT conjugated polymer **d**, CdSe/CdS core-shell quantum dots **e**, $CsPbI_3$ nanocrystals. Inset: Macroscopic image of the metasurface coated with different emitters.

Supplementary References

1. Mendoza-Carreño, J. *et al.* Nanoimprinted 2D-Chiral Perovskite Nanocrystal Metasurfaces for Circularly Polarized Photoluminescence. *Adv. Mater.* **35**, 2210477 (2023).

2. Tang, Y. & Cohen, A. E. Optical Chirality and Its Interaction with Matter. *Phys Rev Lett* **104**, 163901 (2010).

3. Maksimov, A. A. *et al.* Circularly polarized light emission from chiral spatially-structured planar semiconductor microcavities. *Phys Rev B* **89**, 045316 (2014).

4. Ramamurthy, M., Pachidis, P., Cote, B. M. & Ferry, V. E. Circularly Polarized Photoluminescence from Nanostructured Arrays of Light Emitters. *ACS Appl. Opt. Mater.* **1**, 491–499 (2023).

5. Lee, Y.-T. *et al.* Angular Control of Circularly Polarized Emission from Achiral Molecules via Magnetic Dipoles Sustained in a Chiral Metamirror. *ACS Appl. Mater. Interfaces* **15**, 36945–36950 (2023).

6. Seo, I. C. *et al.* Circularly Polarized Emission from Organic–Inorganic Hybrid Perovskites via Chiral Fano Resonances. *ACS Nano* **15**, 13781–13793 (2021).

7. Kitzmann, W. R., Freudenthal, J., Reponen, A.-P. M., VanOrman, Z. A. & Feldmann, S. Fundamentals, Advances, and Artifacts in Circularly Polarized Luminescence (CPL) Spectroscopy. *Adv. Mater.* **35**, 2302279 (2023).

8. Novotny, L. & Hecht, B. *Principles of Nano-Optics*. (Cambridge University Press, Cambridge, 2012). doi:10.1017/CBO9780511794193.

9. Klimov, V. I. Optical Nonlinearities and Ultrafast Carrier Dynamics in Semiconductor Nanocrystals. *J. Phys. Chem. B* **104**, 6112–6123 (2000).
